# Genotype by environment interaction under two production systems utilizing a random regression model for milk production traits in multiparous South African Holstein dairy cattle

**DOI:** 10.1186/s12711-026-01098-x

**Published:** 2026-10-30

**Authors:** Michiel Van Niekerk, Frederick W. C. Neser, Jacobus B. Van Wyk, Vincent Ducrocq

**Affiliations:** 1 Department of Animal Science, University of the Free State, Bloemfontein, 9301 South Africa; 2 Université Paris-Saclay, INRAE, AgroParisTech, UMR GABI, Jouy-en-Josas, 78350 France

## Abstract

**Background:**

Dairy cattle in South Africa (SA) have to produce in varying environments which can roughly be separated as total mixed ration (TMR) and pasture (PAST) production systems according to the level of rainfall in the herd area. Previous studies aimed to determine a possible genotype × environment interaction (GxE) using 305-day lactation records, finding no interaction, but different heritabilities. An alternative random regression (aRR) model was developed using SA Holstein test-day records of herds with known production system used (TMR or PAST, analysed independently). The aRR model separates the animal genetic (additive) effect into a general production level and a linear persistency effect and shows clear differences in the genetic variances and heritability estimates over the lactation between TMR and PAST. The aim of this study was to further investigate the existence of a possible GxE interaction, using comparable data, methods and RR model to differentiate each trait (milk, fat and protein production for the first 3 lactations) separately for the two production systems (TMR or PAST). The possible reranking of sires on resulting estimated breeding values from the analysis between TMR and PAST were also investigated.

**Results:**

Genetic correlations (0.83 to 0.93) of the genetic general production level effect between TMR and PAST were strong, while corresponding correlations of the genetic linear persistency effect were moderate (0.40 to 0.60) for all traits. Significant reranking of sires between TMR and PAST are predicted for genetic linear persistency, as well as genetic total production.

**Conclusions:**

Genetic correlations show a significant genotype by environment interaction for the genetic linear persistency effect between the two production systems in SA Holsteins. Genetic evaluations should analyse production traits in the different systems as genetically different traits, utilizing an applicable random regression model that can effectively dissociate genetic general production level from genetic linear persistency. Estimating separate breeding values for persistency for each production system is justified.

**Supplementary Information:**

The online version contains supplementary material available at https://doi.org/10.1186/s12711-026-01098-x.

## Background

Selection response under different “environments” in the presence of genotype by environment interaction (GxE) can vary substantially. This can be attributed to different genes that may be necessary to adapt and thrive in different environments [1]. South Africa (SA) is a country with diverse environments, classified into 13 distinct climate zones according to the Köppen-Geiger classification method [2]. Despite the varying climatic conditions and regions in SA, [3] found no GxE interaction in SA Jersey cattle for milk production (Milk) in various groups based on different production systems and climatic areas, using a bivariate (lactation based) animal model. However, [4] compared SA dairy cows on a concentrate, mixed or pasture (PAST) ration and showed significant phenotypic differences in production between these feeding systems and speculated about a possible GxE interaction. Various other studies reported mostly minor to no GxE interactions for production traits between some type of control (conventional or confined environment) vs grazing herds [5–8]. It is quite possible that the lack of GxE interactions could have been due to limited differences among environments in each study, as the magnitude of GxE between environments is influenced by, amongst other factors, the amount of variation between the climatic areas or management systems [9].

All these studies used whole lactation (mostly 305-day) records, which prevents the differentiation between production-level and -persistency as shown by [10, 11] and [12]. Previously, [13] evaluated various persistency measures using test-day (TD i.e., test-date) milk yield records. They showed that more persistent cows could be more efficient (and/or profitable) as they require less concentrate to produce the same amount of milk than less persistent cows. According to [14] the concentrate reduction amounts to 3%, with a further 7% reduction in health and reproduction cost. An economic value for persistency of 3.4% relative to the corresponding value of 305-day production at a calving interval of 12.4 months was indicated [15], with a threefold increase in the economic value of persistency when the average calving interval was 13 months. They stated that taking health and reproductive costs into account, the economic importance of persistency will increase further.

Economic values for SA Holstein and Jersey cattle of different payment systems were compiled [16] and included milk volume, fat and protein yield as production traits, as well as live weight, longevity, calving interval and somatic cell score (SCS). However, the effect of lactation persistency per se was not part of the study. The importance of protein yield and (milk) volume, longevity and SCS as traits to be included into breeding objectives for SA Holstein cattle were emphasized [17]. There exists a genetic relationship between all of these traits and persistency. A persistency index for SA dairy breeds based on the Canadian Persistency Index was implemented by [18].

A preliminary study of GxE interaction in SA Holstein was conducted for 305-day production and age at first calving between total mixed ration (TMR) and PAST feeding system herds with no such interaction for milk production [19]. The authors [19] did, however, highlight the possible existence of a GxE interaction for production traits due to the large difference between the heritabilities for the same trait in the two feeding systems. This result was confirmed using a TD, multi-lactation, random regression (RR) model with differing heritability levels and patterns over the lactation for production traits between TMR and PAST [20]. Another study [21] also concluded that an important GxE interaction exists in SA Holstein cattle which could result in reranking of sires between different feeding systems (TMR and PAST). Their study showed that the average annual rainfall of the region in which the herds are kept are a determinant of the production (feeding) system used, and emphasized that GxE interaction research in SA dairy cattle should be expanded by using more complex genetic evaluation models [21]. The current model used for genetic evaluations in SA Holsteins is a TD, multi-lactation, fixed regression model [22], without considering the production system of herds in the analysis.

The objective of this study was to investigate the previous indications of a possible GxE in milk production traits under a TMR versus a PAST production system, specifically using a TD, multi-lactation model based on the alternative random regression (aRR) developed in [20], which analyzes TMR and PAST separately. Subsequent genetic parameters for and between TMR and PAST from such a model can give new insights into the existence and magnitude of a possible GxE as it includes a genetic general (average) production (level; PROD) and genetic linear persistency (slope; PERS) effect. TMR and PAST productions were never analyzed together as different traits to investigate GxE interaction in SA before. The second objective was to consider a potential reranking of sires in the different production systems.

## Methods

### Dataset

TD (*n* = 2 310 430) and pedigree records from all herds (220) participating in milk recording were made available by the South African Holstein Cattle Breeders’ Society for the study. TD records for milk, fat and protein production (Milk, Fat and Protein; kg) of cows with first lactation records, milked 2 or 3 times a day (number of milkings) for the first 3 lactations (Lac1, Lac2 and Lac3, with Lac2 and Lac3 demarcated as “later lactations”) were used. Cows with less than 7 TD records per lactation as well as cows without calving dates were excluded from the dataset. Requiring a minimum of 7 TD records per lactation was deemed necessary to accurately measure persistency. Previous research [20] showed that the persistency component explained a significant proportion of the genetic variance when considering the eigenvalues in Milk Lac1 (27.8% and 25.3% as the second eigenvalue in PAST and TMR, respectively), more so than the comparable study by [23]. The first eigenvalue in [20] represented genetic production level in Lac1 and was 63.5% and 62.8% for PAST and TMR, respectively. Each lactation consisted of TD records that ranged from 6 to 300 days in milk (DIM). Lactations where the first TD record was not within 65 DIM were discarded. Also, lactations that had a TD record interval equalling 100 days or greater were removed, and only one TD interval between 70 and 100 days in a lactation was allowed [22]. The dataset before editing included a large number of cows without any TD records for Lac1, with only Lac2 and/or Lac3 recorded. To minimize bias these cows were discarded.

Contemporary groups (Herdx TD x Number of milkings) had to consist of at least 5 cows. Calving ages of cows (in months) were grouped into 8 calving age classes as follows: Class 1, 20 to 23; Class 2, 24 to 27; Class 3, 28 to 31; Class 4, 32 to 35; Class 5, 36 to 41; Class 6, 42 to 47; Class 7, 48 to 55; Class 8, 56 to 69. The calving month variable followed the natural 12 months of the year, while calving season was also used and followed the natural seasons in South Africa (Summer, Season 1: December, January, and February; Autumn, Season 2: March, April and May; Winter, Season 3: June, July and August; Spring, Season 4: September, October and November). The calving year ranged from 1988 to 2016. Previous calving interval classes were categorized and ranged from 312 to 730 days (Class 1: 312 to 353; Class 2: 354 to 395; Class 3: 396 to 437; Class 4: 438 to 479; Class 5: 480 to 521; Class 6: 522 to 563; Class 7: 564 to 730; Class 8: no previous calving interval – first calf cows). Cows with unknown sires were discarded.

The final dataset consisted of 74 herds with a total of 571 236 TD records utilizing a TMR production system and 22 herds with a total of 215 592 TD records utilizing a PAST production system (Table 1). The total number of sires in the TD dataset was 1 221. By far the largest proportion (53%) of these sires were from North America (United States and Canada). This proportion varied little between TMR and PAST.

**Table 1:** Descriptive statistics for cows with test-day records for production traits in the first 3 lactations in the total mixed ration (TMR) and pasture (PAST) production systems (TD dataset)

	Lac1		Lac2		Lac3	
Item	TMR	PAST	TMR	PAST	TMR	PAST
Avg. Milk (kg)	27.8 (7.8)	22.0 (5.6)	32.0 (9.9)	25.7 (7.5)	33.2 (10.3)	27.6 (8.2)
Avg. Fat (kg)	1.0 (0.3)	0.8 (0.2)	1.2 (0.4)	0.9 (0.3)	1.2 (0.4)	1.0 (0.3)
Avg. Protein (kg)	0.9 (0.2)	0.7 (0.2)	1.0 (0.3)	0.8 (0.2)	1.1 (0.3)	0.9 (0.2)
*n* records	355 361	117 115	160 158	65 011	55 717	33 466
*n* cows	45 583	14 947	20 475	8 269	7 155	4 277
*n* groups	8 437	3 144	5 993	2 401	3 329	1 685
… with 2 milkings	4 527	2 958	3 149	2 273	1 749	1 615
… with 3 milkings	3 910	186	2 844	128	1 580	70
*n* records^1^ / cow	7.9	7.9	7.9	7.9	7.9	7.9
*n* records^1^ / group	95	53	56	51	34	30
*n* sires	1 172	760	1 119	639	879	520
*n* sires-5	1 115	560	877	404	410	258
*n* shared sires-5	454		297		154	
*n* sires-20	600	221	300	115	78	53
*n* shared sires-20	168		82		24	
*n* daughters^1^ / sire	39	20	18	13	8	8

Standard deviations in parentheses; Laci = Lactation i; Avg. = Average; Milk = Milk production; Fat = Fat production; Protein = Protein production; *n* = number of; Superscript 1 = average number of; group(s) = contemporary group defined by the Herd ×Test-day ×Number of milkings interaction effect; milkings = Number of milkings per 24 hours; sires-5 = sires with a minimum of 5 daughters (with records); shared sires-5 = sires with a minimum of 5 daughters (with records) in the pasture and total mixed ration production systems each; sires-20 = sires with a minimum of 20 daughters (with records); shared sires-20 = sires with a minimum of 20 daughters (with records) in the pasture and total mixed ration production systems each

Table 1 shows the total number of sires and the number of sires with at least 5 daughters per lactation in TMR and PAST. There were 711 shared sires between TMR and PAST with an average of 49 and 20 daughters in the two production systems, respectively. Out of these, 454 (64%) had at least 5 daughters in both TMR (average of 63) and PAST (average of 28) for Lac1. These sires are termed shared sires-5 (Table 1). Likewise, the more influential sires that had at least 20 daughters in both TMR (average of 112 daughters) and PAST (average of 55 daughters) were 168, and are termed shared sires-20.

The pedigree dataset (123 082 animals) used in the analyses consisted of 1 221 sires, all with progeny, 42 409 dams with only progeny, and 19 572 dams with records and progeny (total dams = 77 399). The numerator relationship matrix was calculated to form the (pedigree based) A-inverse matrix incorporated in the mixed model analyses (Table 2). Animals in the pedigree dataset stretched over ~15 generations (averaged over sires and dams).

**Table 2:** Description of the random regression (RR) model used to analyse milk, fat and protein production

Fixed effects (categorical)	Fixed regressions on DIM	Random regressions on DIM
Herd x TD x Number of milkings per day (HTMi)	Herd	Animal Genetic (additive; AG)
	Year^1^ x Lactation	Permanent Environmental (PE)
Herd x Year x Season^1^ x Lactation (HYSL)	Month^1^	Heterogeneous residual variance (10 classes)
Age^1^ (months) x Lactation	Calving interval (previous) (8 classes)	
	Age^1^ (8 classes)	

TD = Test-date; DIM = Days in milk. ^1^ = at calving i.e., calving year, calving season, calving age etc.

### Model for analysis

Milk was first analysed to test the significance of fixed effects. The initial model comprised of the two categorical fixed effects of Herd x TD x Number of milkings (HTMi), and Herd x Calving Year x Calving Season x Lactation (HYSL), while also including Herd-specific fixed regressions on DIM to adjust for environmental variations affecting the shape of the lactation curve. Calving Year x Lactation and Calving Month fixed regressions on DIM were included in the same way as Herd, as part of the first model. Including the HYSL fixed effect in the model will adjust records for stage of lactation where the cows in a group calved within the same ~90-day window. Next, a calving age effect was added to the model and tested in various ways: as covariate in days or months, or categorically in months, or as an interaction effect with Lactation (number). Additionally, the effects of calving age classes were tested as fixed regressions on DIM. Lastly, the previous calving interval effect were tested as a covariate and as calving interval classes fixed regressions on DIM.

The Bayesian Information Criteria (BIC = 2logL + log(N)*k, where logL denotes the value of the maximum log likelihood of the model, N is the sample size of the TD dataset, and k is the number of parameters in the model) was used to assess the differences in goodness of fit between the different models.

Milk, Fat and Protein were analyzed separately, using restricted maximum likelihood (REML) in Wombat [24]. Here, a TD, multi-lactation (first 3 lactations) RR model was used to analyse each of the traits to determine the (co)variance components and subsequent heritabilities, genetic correlations and EBVs. Furthermore, Legendre polynomials to the order of 4, 5 and 6 were tested as regressions on DIM within various fixed effects (Table 2) for Milk lactation (Lac) 1, 2 or 3. Natural splines with 6 knots (DIM 6, 20, 50, 135, 245, 300) were also evaluated as a regression function. Previously, [20] demonstrated that this model provided a superior fit to the data based on BIC, outperforming the Wilmink curve [25], Legendre polynomials (orders 3 and 4), and both cubic and natural splines with four knots.

The residual variance was considered as heterogeneous and TD records were assigned to 9 residual effect classes of 30 days each, and 1 class of 25 days at the end of the lactation (10 classes in total; 295-day lactation). The final model used is described in Table 2. Additive Genetic (AG) and Permanent Environmental (PE) effects were assumed normally distributed with an average of 0, and were fitted in the analyses as random regression effects. The AG and PE effects described each lactation separately, and each effect consisted of a first order regression on DIM, yielding two components for both effects: an intercept (a_0_) and a slope (a_1_). In the case of the AG effect, the intercept represents the general (additive) genetic production level (PROD) effect. This effect is equivalent to genetic average production across the lactation due to the centering of the time covariates i.e., DIM. The regression slope (DIM were standardized from 0.5 for DIM 6, to −0.5 for DIM 300 for each lactation) describes the genetic (linear) persistency (PERS) effect per lactation (DIM 6 to 300). Standardizing DIM from 0.5 to −0.5 is deliberate to define the natural drop (negative trajectory) in production and simplify the variance multiplier in Equation 3 to exactly 1. The PROD and PERS effects yield 6 AG components in total over the 3 lactations per production system i.e., the AG (co)variance matrix has a dimension of 6 × 6. Together these components (PROD and PERS) represent genetic total production (TOTAL). TD records obtained either in a TMR, or a PAST production system were treated as two separate traits, and were analysed together in a multiple trait analysis resulting in 12 AG components i.e., the AG (co)variance matrix of dimension 12 × 12. The AG (co)variance matrix was transformed to a 1770 × 1770 matrix (1770 = 295 DIM × 3 lactations × 2 production systems; **AG**_**T**_): **AG**_**T**_ = **ΦAGΦ’**, where **Φ** is a matrix with covariates representing the standardized DIM values (1770 × 12 matrix). The same method was applied to calculate the corresponding PE_T_ matrix.

Heritability estimates ($h_t^2$) for TOTAL at any given DIM *t* in a given lactation in TMR or PAST can be calculated as:

1 $$h_t^2 = {V_{A{G_t}}} / {V_{A{G_t}}} + {V_{P{E_t}}} + {V_{{e_c}}},$$

where ${V_{A{G_t}}}$ and ${V_{P{E_t}}}$ are the variances of the AG and PE random effects, respectively, and are obtained from the corresponding AG_T_ and PE_T_ matrices. ${V_{{e_c}}}$ is the residual variance of class *c* corresponding to day *t*. Considering the definition of the PERS component, the heritability estimates for the PROD component ($h_{PROD}^2$) was calculated using the midpoint of the lactation (DIM 153) where the linear slope covariate becomes 0. This approach eliminates all covariance math. The equation for a given lactation in TMR or PAST can be calculated as:

2 $$h_{PROD}^2 = {V_{A{G_{PROD}}}} / {V_{A{G_{PROD}}}} + {V_{P{E_{PROD}}}} + {V_{{e_5}}},$$

where ${V_{A{G_{PROD}}}}$ and ${V_{P{E_{PROD}}}}$ are the PROD variances from the corresponding AG and PE (co)variance matrices, and ${V_{{e_5}}}$ is the residual variance for class 5 to which DIM 153 belongs. Heritability estimates for the PERS component ($h_{PERS}^2$) were calculated as the total drop in production from DIM 6 to 300. The difference vector (**d**) to determine the difference in production between these DIMs removes the intercept, with a resulting linear multiplier of −1:

3 $${\mathbf{d}} = {z_{300}}\, - {z_6} = [1,\, - 0.5] - [1,\,0.5] = [0,\, - 1.0],$$

where z is the standardized covariate (DIM). The variance of this difference is (−1)^2^ = 1, and thus the AG and PE variances for PERS are multiplied by 1. Assuming temporary environmental residuals across different DIM are mutually independent in a RR TD model, the variance of a difference between two such variables is equal to the sum of their individual variances. Consequently, the residual variance for PERS (${V_{{e_{PERS}}}}$) is a function exclusively of the residual variances at the start (DIM 6) and end (DIM 300) points, as shown in Equation 4.

4 $${V_{{e_{PERS}}}} = {V_{{e_{DIM \,6}}}} + {V_{{e_{DIM \,300}}}},$$

The equation for $h_{PERS}^2$ pertaining to a certain lactation in TMR or PAST can be presented as:

5 $$h_{PERS}^2 = {V_{A{G_{PERS}}}} / {V_{A{G_{PERS}}}} + {V_{P{E_{PERS}}}} + {V_{{e_1}}} + {V_{{e_{10}}}},$$

where ${V_{A{G_{PERS}}}}$ and ${V_{P{E_{PERS}}}}$ is the PERS variances from the corresponding AG and PE (co)variance matrices. ${V_{{e_1}}}$ and ${V_{{e_{10}}}}$ is the residual variance for class 1 to which DIM 6, and class 10 to which DIM 300 belong, respectively.

The PROD and PERS AG effects result in estimated breeding values (EBVs) for both components (i.e., EBV-PROD and EBV-PERS) for each animal. Hence, over the 3 lactations per production system, AG components 1 to 6 would be EBV-PROD-Lac1, EBV-PERS-Lac1, EBV-PROD-Lac2, EBV-PERS-Lac2, and EBV-PROD-Lac3 and EBV-PERS-Lac3, in that order for each trait (Table 3). These AG components were the same as the ones derived by [20]. The sum of these two components is thus the total production EBV i.e., EBV-TOTAL per lactation.

**Table 3:** Animal genetic components and the production systems, lactations and EBVs they represent in the TMR-PAST model

AG component	Production system	Lactation	EBV-
1	TMR	1	PROD
2	TMR	1	PERS
3	TMR	2	PROD
4	TMR	2	PERS
5	TMR	3	PROD
6	TMR	3	PERS
7	PAST	1	PROD
8	PAST	1	PERS
9	PAST	2	PROD
10	PAST	2	PERS
11	PAST	3	PROD
12	PAST	3	PERS

AG = Animal Genetic (additive); EBV- = Estimated Breeding Value (for production level or persistency); PROD = production level; PERS = persistency

This means that the 12 AG components represent the EBVs as shown in Table 3 for this TMR-PAST analysis (model).

The genetic correlations between TMR and PAST over the first 3 lactations were calculated using the AG_T_ (1770 × 1770) matrix. The 12 × 12 AG (co)variance matrix is also used to calculate the genetic correlations between the different PROD and PERS effects i.e., genetic production level and genetic persistency within and between the lactations.

## Results and discussion

### Model selection and comparison

Analyzing Milk, including calving age in months as a classified fixed effect rather than a covariate (days or months) resulted in a significantly better BIC (improvement of 873 and 941, respectively). Including it with lactation (number) as an interaction effect caused the BIC to improve further by 93 compared to calving age (months) as a classified effect on its own. This calving age x lactation effect also accounted for the confounding effect between these two variables. Also, fitting a calving age class as a fixed regression over the lactation resulted in an additional improvement of 487 in the BIC. Similarly, replacing (previous) calving interval as a covariate with calving interval classes as fixed regressions over DIM resulted in a BIC improvement of 65.

The natural splines with 6 knots were the best fit to describe the fixed regressions over DIM. The improvement in BIC over Legendre polynomials to the order of 6, 5 and 4 were 3 042, 4 568 and 9 820 compared to the splines.

All fixed effects included in the model (Table 2) were found to be significant (*p* < 0.05) and led to a significant improvement in the Bayesian Information Criterion (BIC). Although simpler contemporary group structures are often favored in large-scale national evaluations, a more comprehensive fixed effect structure was required to prevent environmental confounding with genetic effects. This was especially important for the objectives of the study in estimating accurate genetic parameters.

Specifically, the inclusion of the fixed regression of herd on DIM (Herd x DIM) accounted for distinct herd-level management trajectories over the course of lactation. Failing to account for these specific herd curves have been shown to artificially inflate genetic variances at the lactation boundaries [26] and overlook peak-yield variance structures [27].

Furthermore, incorporating the nested categorical effect of HYSL alongside the HTMi effect followed the framework described by [28]. Given the diverse geographic, climatic, and seasonal management practices characteristic of South African dairy systems, the HYSL effect captured macro-environmental and seasonal influences unique to calving blocks within herds that the localized HTMi effect could not fully account for. The inclusion of HYSL resulted in a significant reduction in BIC (−33 552), confirming that the model was statistically optimal and preventing the overestimation of genetic variances.

The use of an intercept and slope (the first and second principal components) to describe the random effects variances was chosen based on the study of [20]. Eigenvalues (%) from their results, using a comparable dataset showed that the first (PROD) and second (PERS) principal components (62.8% and 25.3%, respectively) together explained 88.1% of the genetic variance in TMR, and 91.3% (63.5% and 27.8%, respectively) in PAST for Milk in Lac1. The total genetic variance explained were 98.8% (adding 7.7% and 3.0%, respectively) for TMR, and 99.5% (adding 6.8% and 1.4%, respectively) for PAST, respectively (98.8% to 99.7% for all production traits) after Lac2 is considered in the same way in a multi-lactation analysis. These results are in accordance with other authors [12, 23]. In this study (analysing TMR and PAST together), TMR Lac1 PROD and PERS explained 47.2% and 29.0% (total of 76.2%), and TMR Lac2 PROD and PERS explained 13.9% and 4.7%, bringing the total genetic variance explained to 94.8%.

### Genetic and other variances for milk production

**Fig. 1:**
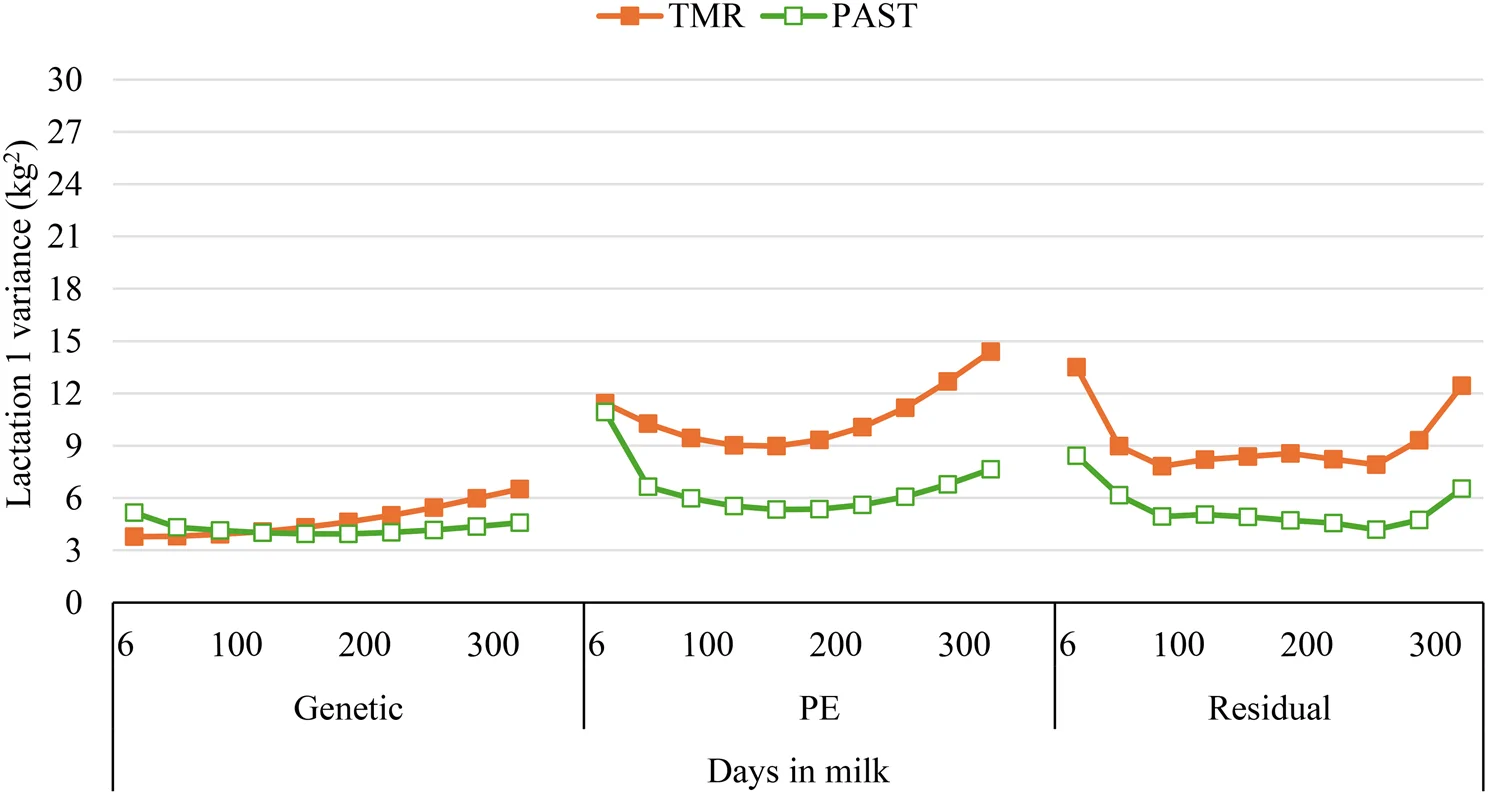
Lactation 1 genetic, permanent environmental and residual variances over days in milk for milk production. TMR = total mixed ration production system; PAST = pasture production system; PE = permanent environmental

**Fig. 2:**
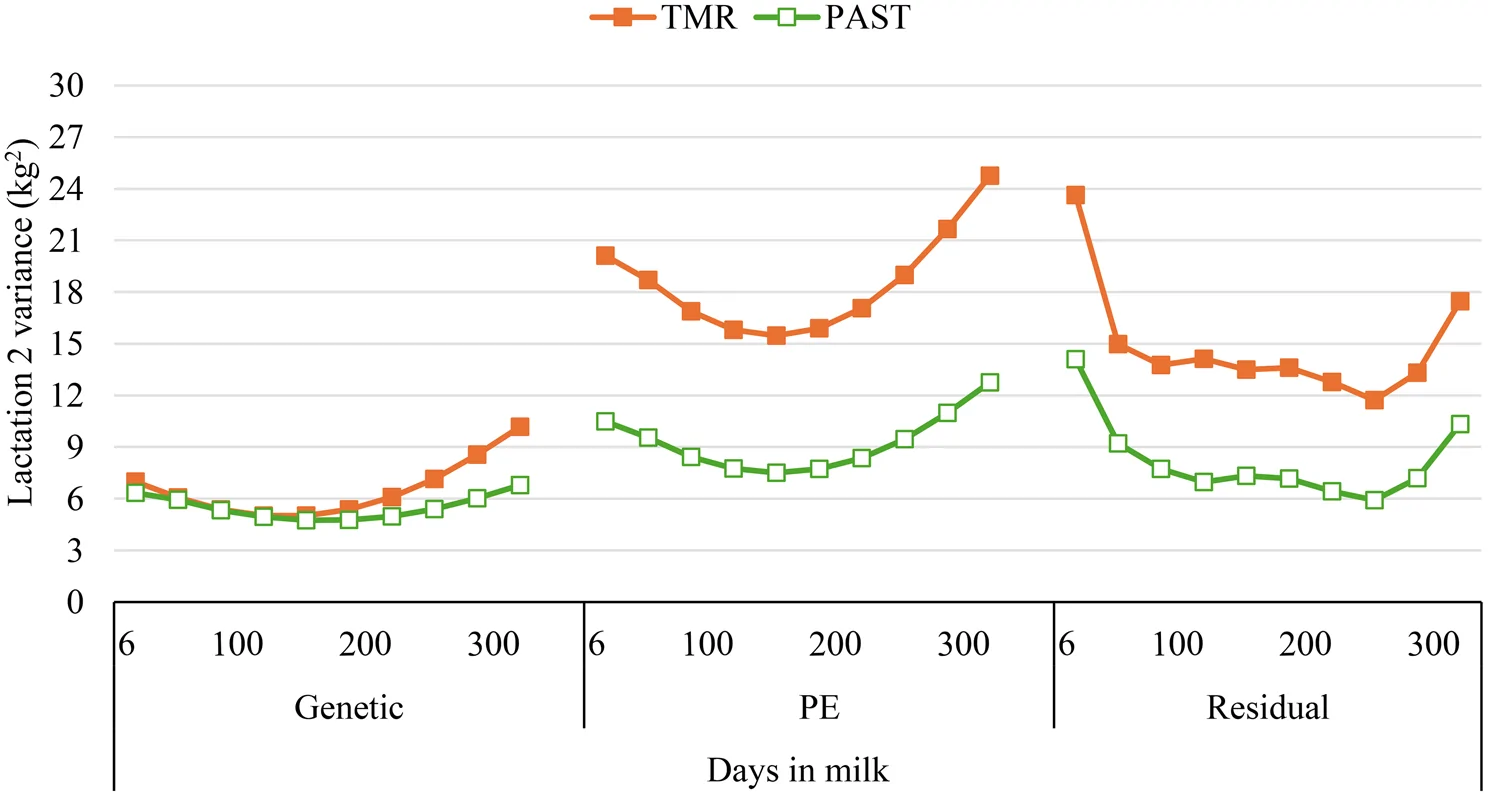
Lactation 2 genetic, permanent environmental and residual variances over days in milk for milk production. TMR = total mixed ration production system; PAST = pasture production system; PE = permanent environmental

**Fig. 3:**
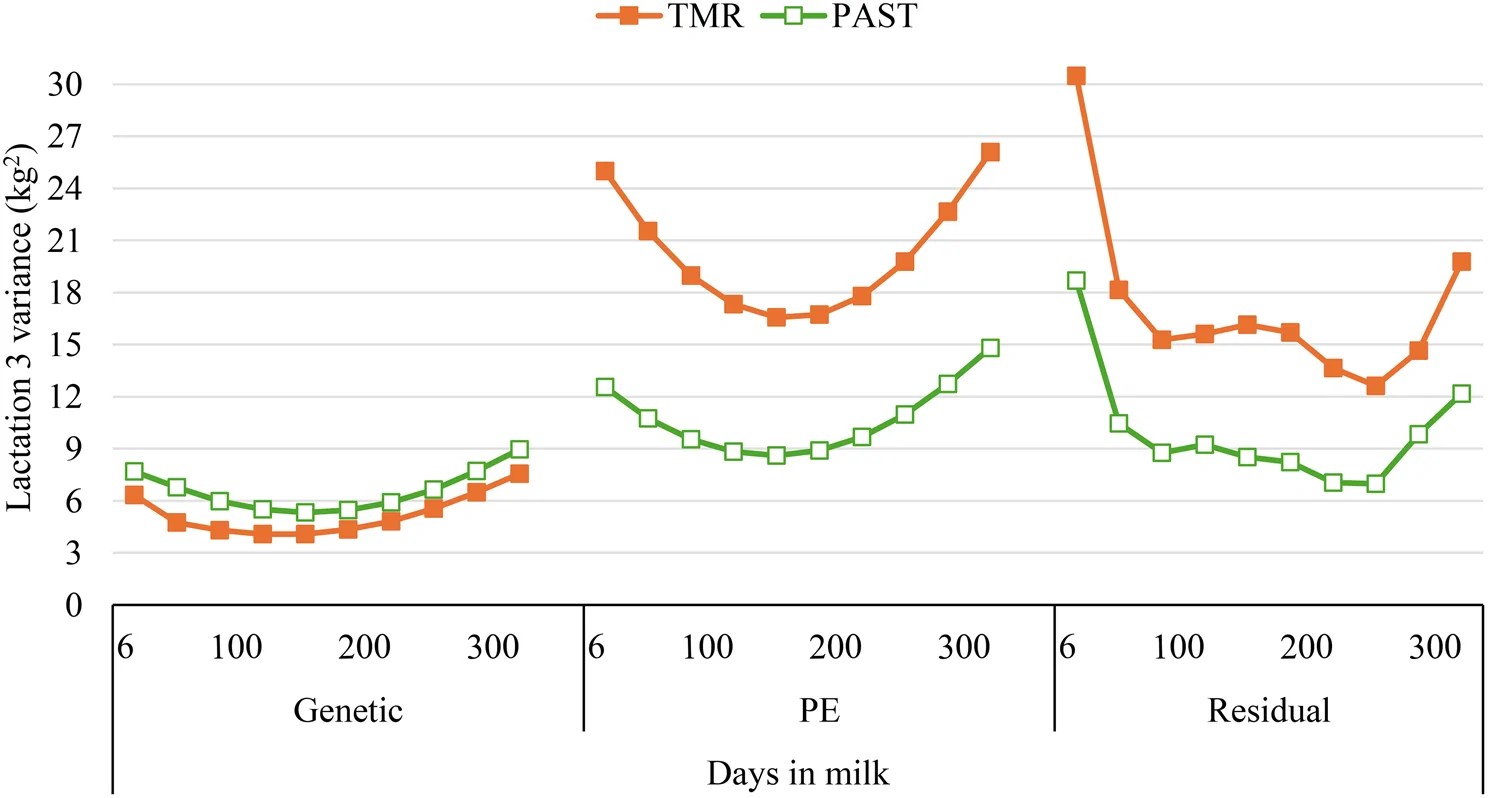
Lactation 3 genetic, permanent environmental and residual variances over days in milk for milk production. TMR = total mixed ration production system; PAST = pasture production system; PE = permanent environmental

The genetic, permanent environmental and residual variance components (TOTAL) of TMR and PAST over DIM 6 to 300 in each of the first 3 lactations for Milk are presented in Figs. 1, 2 and 3, respectively. Corresponding variance components for Fat and Protein are presented in Additional file 1 Fig. S1 to S3 and Additional file 2 Fig. S4 to S6, respectively. TMR genetic variances (Milk) were higher than corresponding PAST genetic variances for all lactations, with the largest differences being in Lac1 and Lac2.

The difference in the shape of the genetic variance curves over DIM (Milk) between TMR and PAST supports the significant GxE interaction. This is especially applicable to the PERS effect considering that the TMR genetic variance increases continuously upwards from PAST from the beginning to the end of Lac1 and especially from DIM 200 onwards in Lac2. Comparably, PAST has a flatter curve over the lactation, especially for Lac1. At the end of Lac1 and Lac2 the TMR genetic variance was 42 and 50% higher, respectively compared to PAST. The differing genetic variance curves between TMR and PAST as Lac1 and Lac2 progress indicate that the GxE interaction is not merely a scale effect. This tendency, especially during late lactation, clearly shows that the genetic ability to produce varies increasingly over the lactation between the two production systems.

Differing random regression coefficients were reported [12] for Milk that described the curves of the genetic effects over the lactation between intensive management (Canada and Italy) and rotational grazing systems (Australia and New Zealand). The (additive) genetic variances for Milk over lactations also differed among low-, medium- and high-production systems with regards to their shape as shown by [29]. Moreover, our results are in accordance with [12] and [29] who also showed the tendency of the genetic variance to be higher in the more optimal environment (TMR in our study). Similarly, [30] showed increasing genetic variance as the environment improves. Genetic differences are more detectable in more favourable environments as animals are more able to express their full genetic merit [31]. In this regard, the environmental variances (PE and residual) indicate that TMR can be considered a more optimal production system compared to PAST for production.

The differing genetic variances over the lactation, within and between TMR and PAST can be explained by cows’ dissimilar genetic energy-balances and nutrient partition profiles, postpartum. Body condition score (BCS) can be used to predict total body energy content (lipid and protein) change over the lactation [32]. Increasing genetic variance for BCS was shown as lactation progressed [33], illustrating genetic differences in cows’ capability to regulate the utilisation of body reserves, especially at the end of the lactation.

### Heritability estimates, permanent environmental and residual variance ratios for milk production

**Fig. 4:**
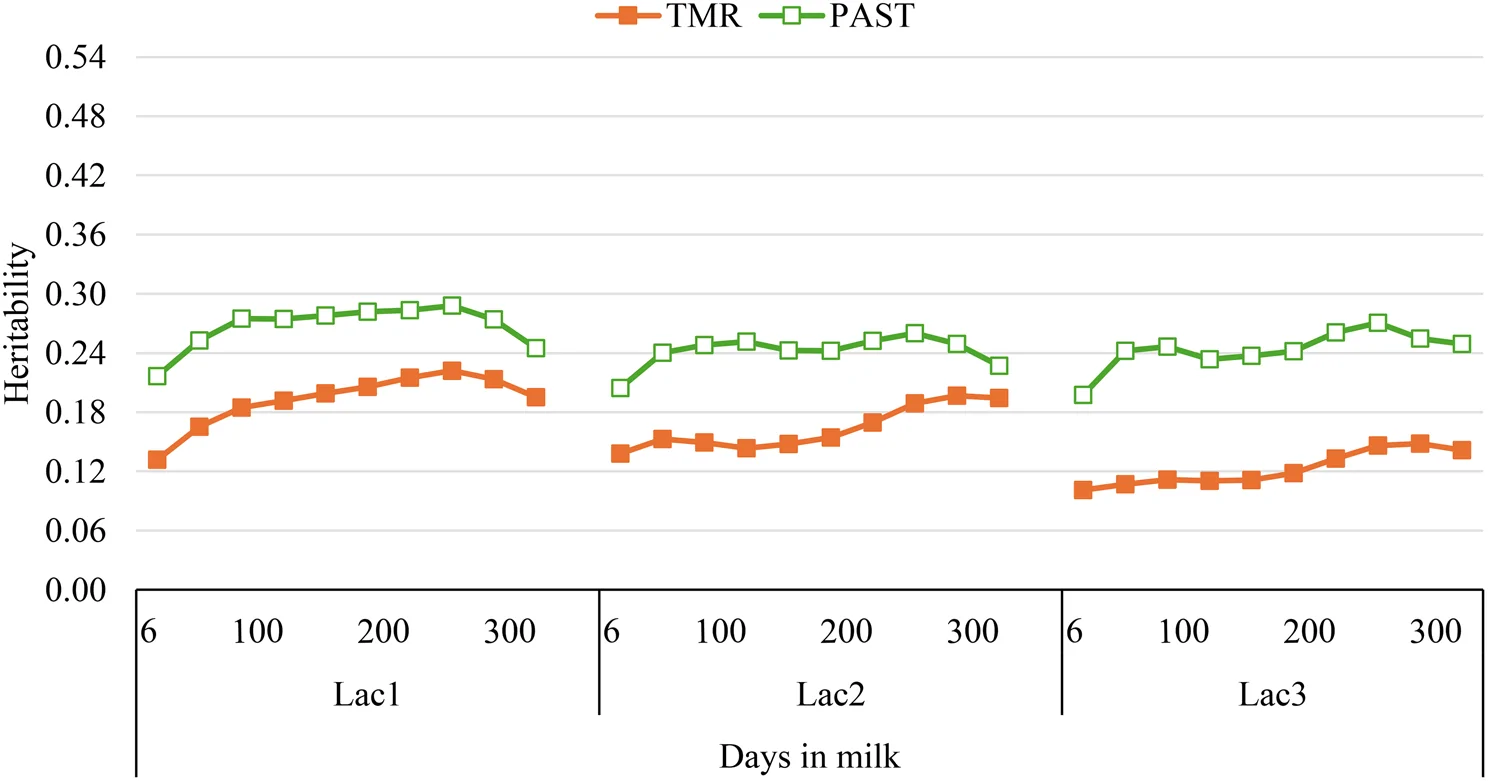
Heritability estimates over days in milk for TOTAL milk production. TMR = total mixed ration production system; PAST = pasture production system; Laci = lactation i; TOTAL = total production i.e., production level (PROD) and persistency (PERS); standard errors for estimates ranged from 0.01 to 0.02, and 0.01 to 0.03 for TMR and PAST, respectively

PAST heritability estimates (TOTAL) were higher than TMR estimates throughout all lactations (Milk; Fig. 4), more so during the first 200 days in Lac1 (average of 0.27 vs 0.18, respectively), Lac2 (average of 0.24 vs 0.15, respectively) and Lac3 (average of 0.24 vs 0.11, respectively). The biggest difference between PAST and TMR heritability estimates were during Lac3 (average of 0.24 vs 0.14, respectively). Corresponding estimates for Fat and Protein are shown in Additional file 3 Fig. S7 and Additional file 4 Fig. S8, respectively.

Average heritability estimates (TOTAL) for Milk, Protein and Fat in PAST and TMR were essentially the same as those reported by [20] in separate analyses of PAST and TMR using the same model, but without the calving age x lactation interaction fixed effect. PAST heritability estimates (0.27 for Lac1) for Milk were closest to estimates from various authors in primiparous dairy cows on a 305-day milk yield basis, with an estimate of 0.19 from [34] and 0.15 from [35]. These two authors reported heritability estimates of 0.42 for the high-input system [34; Luxembourg] and 0.27 for the “fed in confinement” system [35]. Overall, heritability estimates in this study are similar to Holsteins kept in a tropical environment [36] and somewhat higher than [37] and [38].

**Fig. 5:**
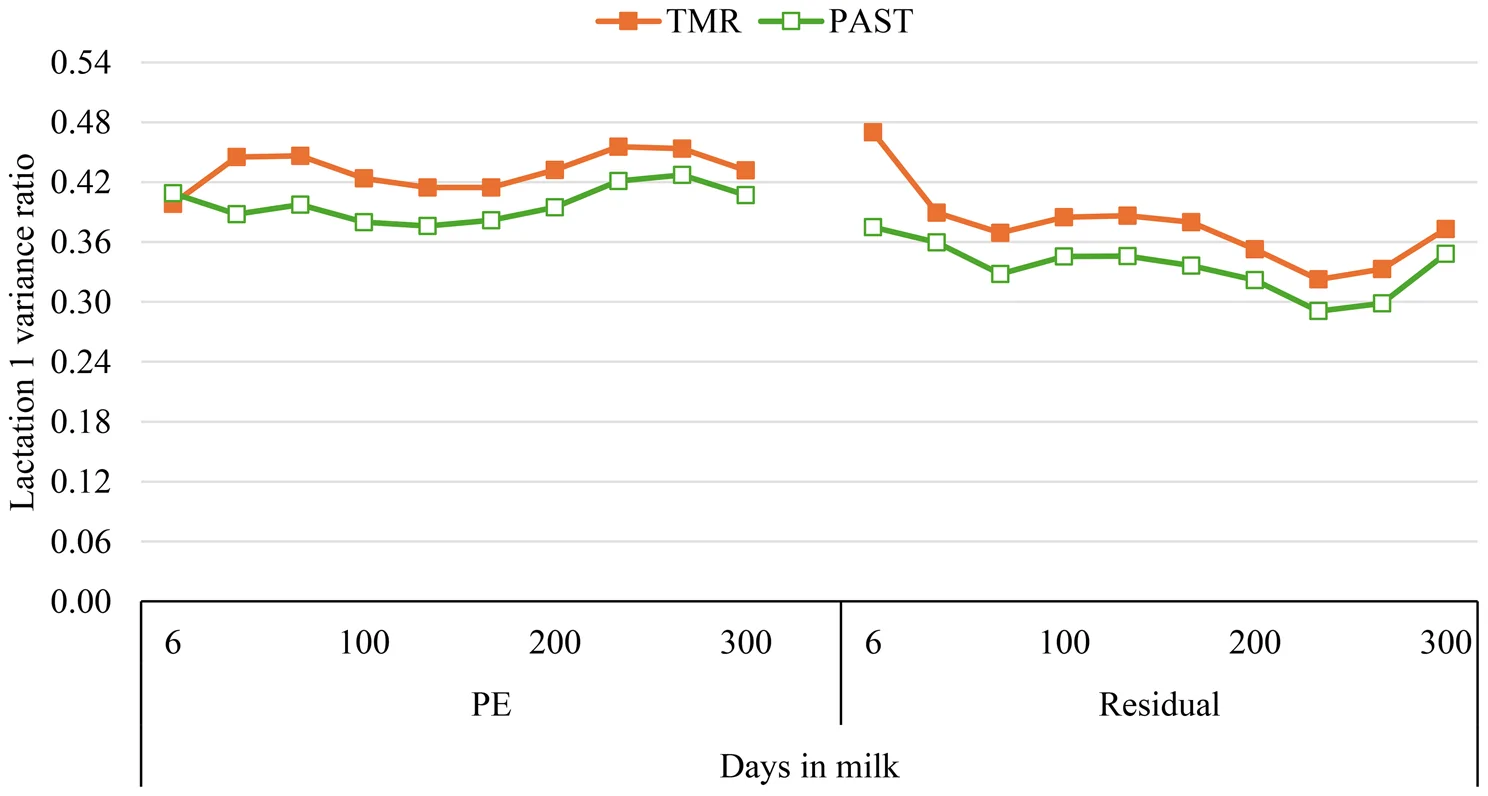
Lactation 1 permanent environmental and residual variance ratios over days in milk for milk production. TMR = total mixed ration production system; PAST = pasture production system; PE = permanent environmental; standard errors for the permanent environmental ratio estimates ranged from 0.01 to 0.02, and 0.01 to 0.03 for TMR and PAST, respectively

**Fig. 6:**
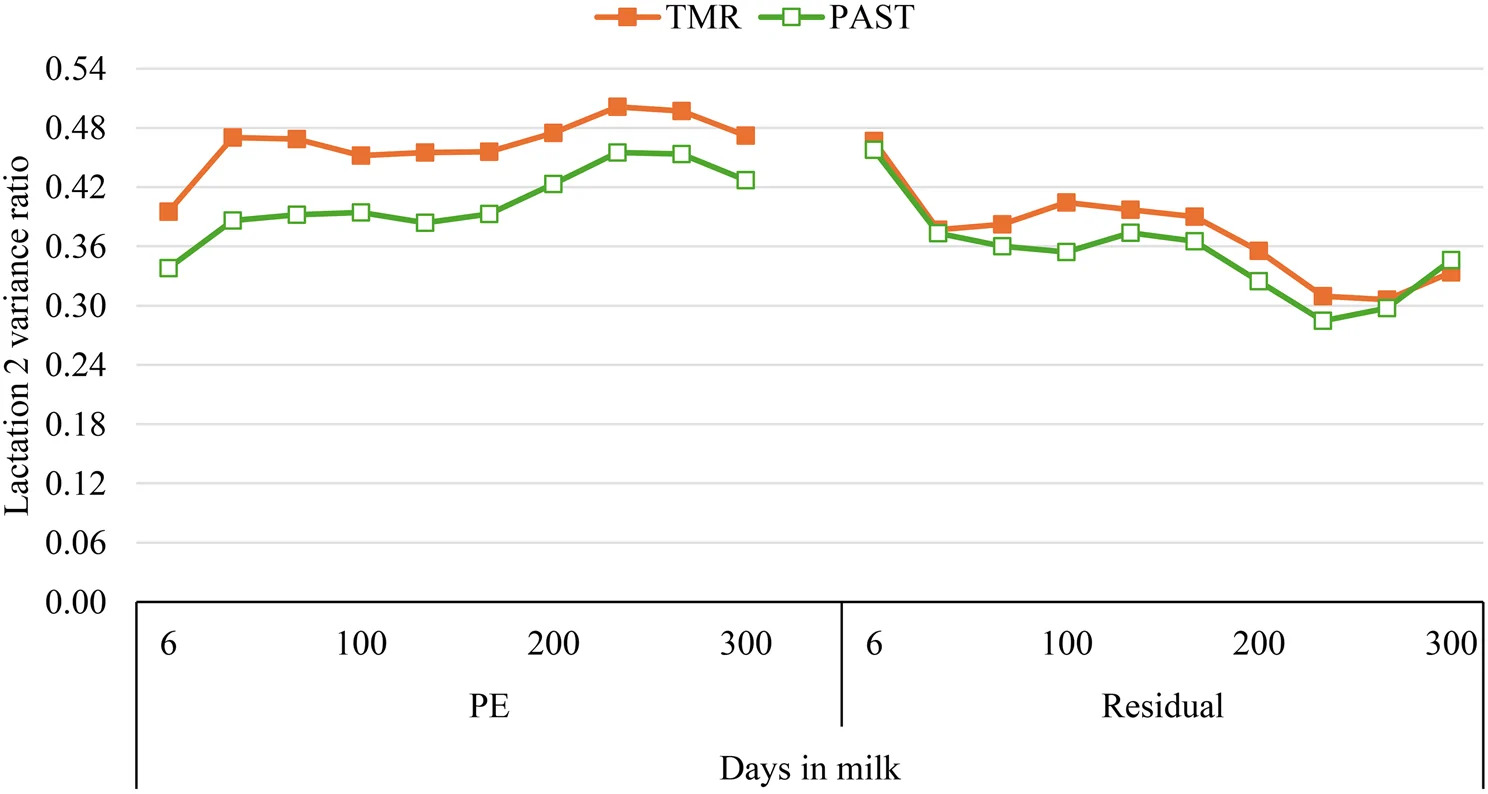
Lactation 2 permanent environmental and residual variance ratios over days in milk for milk production. TMR = total mixed ration production system; PAST = pasture production system; PE = permanent environmental; standard errors for the permanent environmental ratio estimates ranged from 0.01 to 0.02

**Fig. 7:**
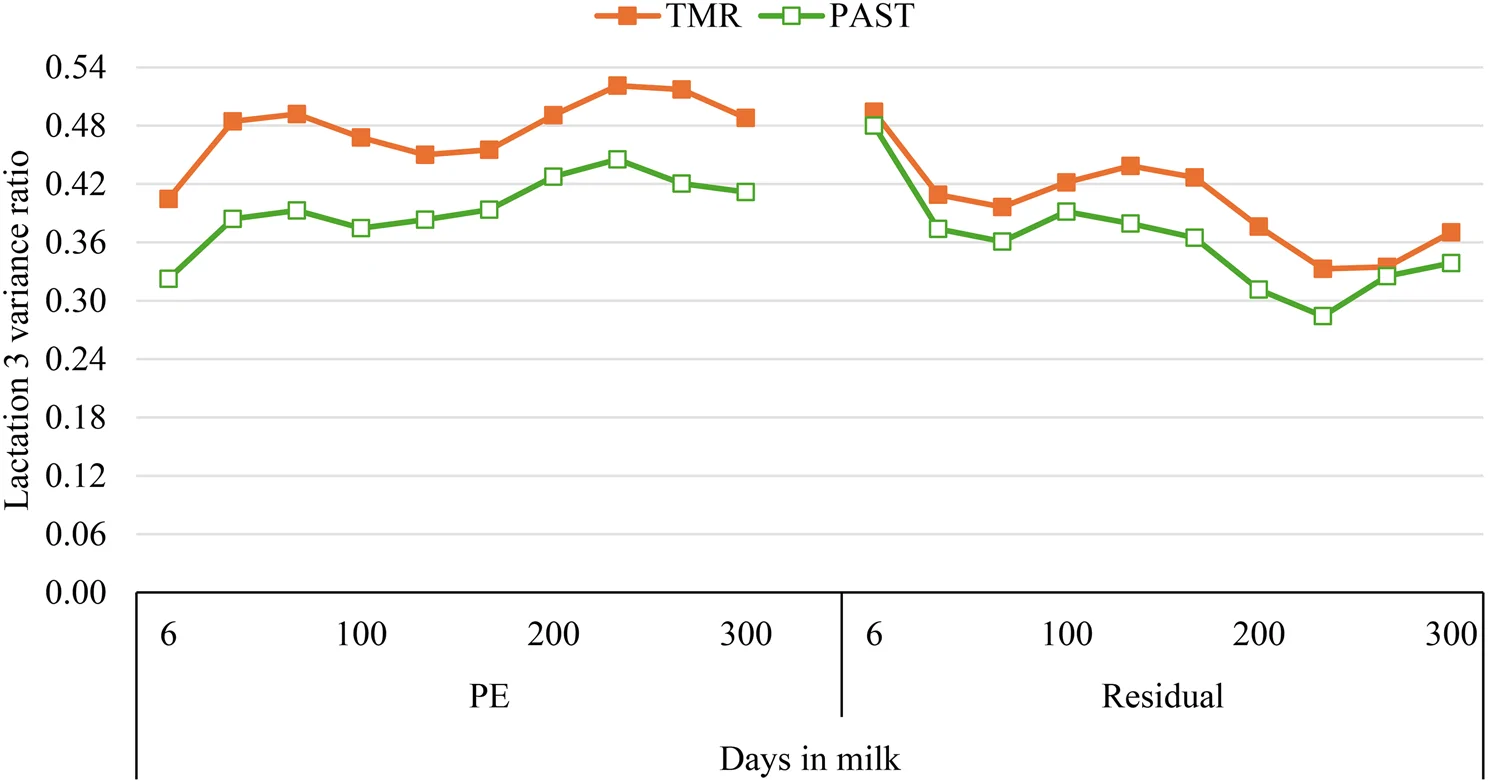
Lactation 3 permanent environmental and residual variance ratios over days in milk for milk production. TMR = total mixed ration production system; PAST = pasture production system; PE = permanent environmental; standard errors for the permanent environmental ratio estimates ranged from 0.01 to 0.02

The higher Milk (and Protein) heritability estimates (Fig. 4) in PAST compared to TMR were mostly because of the higher proportion of the total (phenotypic) variance that was explained by the PE variance (ratios; Figs. 5, 6, 7) in TMR (on average) with a resulting drop in the genetic variance. The same corresponding results with regards to the PE and residual variance (ratios; Figs. 5 to 7 for the residual ratios in this study) were shown by [20] as reasons for differing TMR and PAST heritability estimates. This higher PE ratio tendency in TMR estimates is most likely because TMR cows are fed equivalent to each cow’s milk yield, most probably up to ~ 100 to 150 DIM. This information is not available to incorporate in genetic analyses and accordingly, genetic evaluations cannot accurately distinguish the level of production as being the result of the AG or the individual nutrition level effect. Whilst in PAST, cows have an equal chance to be exposed to the same nutritional level. The study of [35] also implied a higher PE ratio for confined vs grazing herds while [12] reported a higher PE variance for more intensive production systems (Canada and Italy) vs rotational grazing systems (New Zealand and Australia).

Different from Milk, the most important cause of the higher Fat PAST heritability estimates compared to TMR were on average the smaller residual variance ratios in PAST with a resulting increase in the heritability. In the same way, the smaller PE variance ratios in PAST, mostly in later lactations also contributed to the genetic variance explaining more of the total variance.

The corresponding PE and residual variance ratios for Fat and Protein are shown in Additional file 5 Fig. S9 to S11 and Additional file 6 Fig. S12 to S14, respectively.

**Fig. 8:**
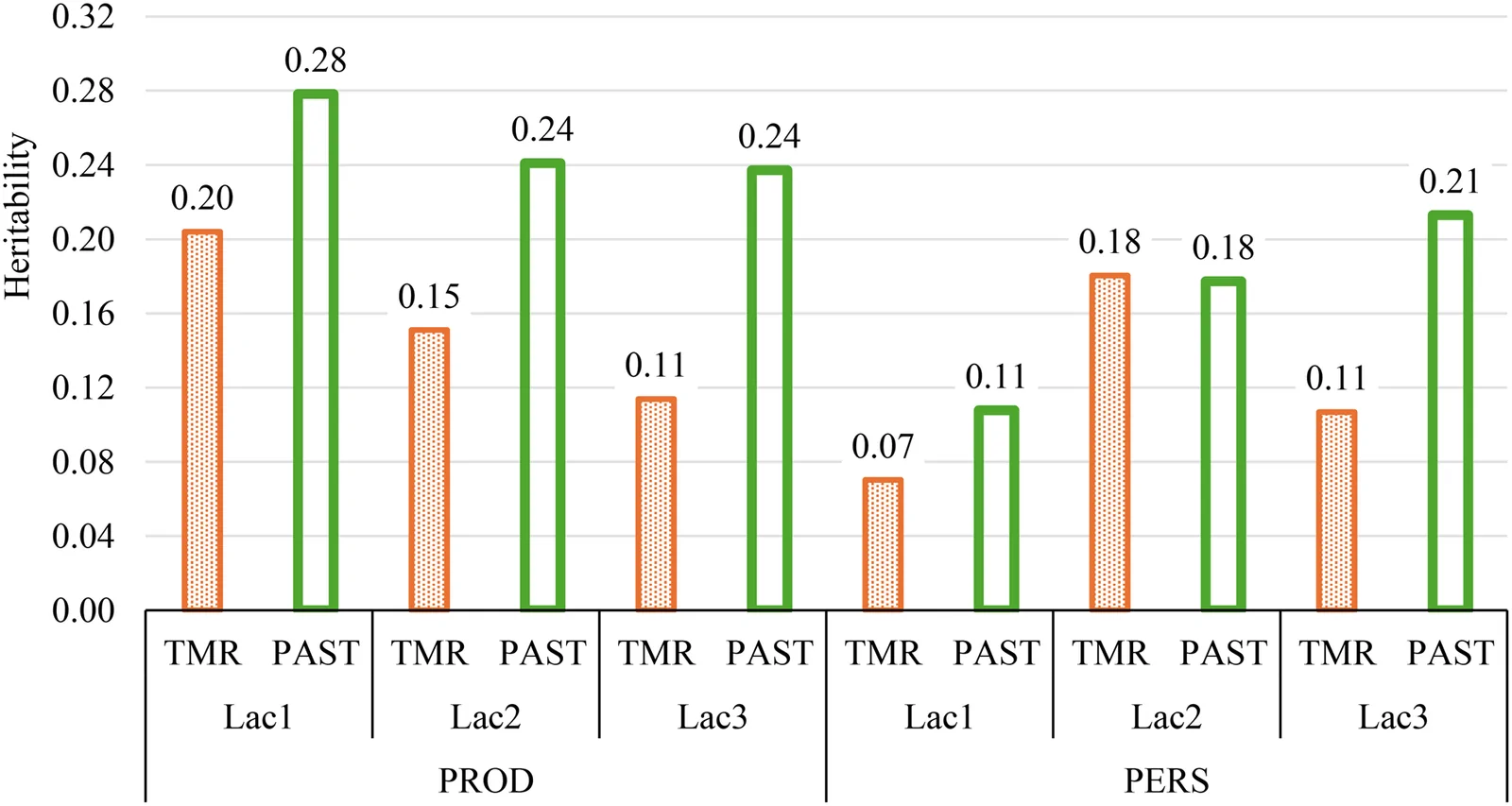
Heritability estimates in the first 3 lactations for production level and persistency of milk production. PROD = production level; PERS = persistency; TMR = total mixed ration production system; PAST = pasture production system; Laci = lactation i

Heritabilities for TMR PROD Lac1 were between low and moderate for Milk (Fig. 8) and Protein, while the corresponding estimate for Fat were low (0.20, 0.17 and 0.10 for Milk, Protein and Fat, respectively). Fat and Protein estimates are shown in Fig. S15 and S16 in Additional files 7 and 8, respectively. Corresponding PAST heritabilities were higher with estimates of 0.28, 0.22 and 0.17 for Milk, Protein and Fat, respectively. PROD later lactation heritabilities were lower than Lac1 for all traits, but more so in TMR (0.07 to 0.15) than in PAST (0.14 to 0.24). This pattern can be explained by the smaller change in the genetic variances from Lac1, to Lac2 and Lac3, compared with the larger corresponding change in the phenotypic variances (i.e., −6 and 15% compared to 56 and 68% in TMR, and 20% and 36% compared to 39% and 60% in PAST for Milk; not shown). Correspondingly, Fat and Protein estimates followed the same pattern.

TMR PERS Lac1 heritabilities for all traits (Fig. 8 for Milk; Fig. S15 and S16 in Additional file 7 and 8 for Fat and Protein, respectively) were lower (0.04 to 0.07) compared to PROD Lac1. Similar to PROD, Lac1 PERS heritabilities in PAST were higher (0.06 to 0.11) than in TMR. In contrast to PROD, PERS heritabilities for later lactations were higher than for Lac1 in TMR (0.08 to 0.18), and PAST (0.10 to 0.21). These results are attributable to the significant increase in the genetic variances for PERS (not shown) from Lac1, to Lac2 and Lac3, relative to the lower corresponding increase in the phenotypic variances (i.e., 405% and 234% compared to 97% and 120% in TMR, and 213% and 373% compared to 90% and 139% in PAST for Milk; not shown). Similarly, Fat and Protein estimates followed the same tendency.

The observed increase in PERS heritability from the first to later lactations (0.08 to 0.18 in TMR; 0.10 to 0.21 in PAST) is strongly supported by early random regression literature. A study by [39] similarly found that heritabilities for distance-based persistency increased from ~0.08 in parity 1, to 0.20 in parities 2 and 3, driven by a significant increase of genetic variance as cows mature. This aligns with [40] who reported persistency heritabilities of 0.05 to 0.14 using comparable point-to-point (DIM) definitions of persistency. Under a similar definition, [38] also reported lower first-lactation persistency heritabilities compared to later lactations, with their overall estimates across the first 3 lactations ranging from 0.10 to 0.20.

While pasture-based (PAST) heritabilities in our study were consistently higher than TMR, likely due to differences in residual variance scaling at the DIM 6 and 300 anchor points, this parity-driven variance trajectory remains consistent across production environments. Furthermore, similar to our results in Lac1, [38] reported higher heritabilities for PROD than for PERS in milk and protein yield, with their PROD estimates (0.11 to 0.18) aligning closely with our TMR values, but with lower heritabilities than our PAST estimates.

### Genetic correlations within lactations between TMR and PAST for milk production

**Fig. 9:**
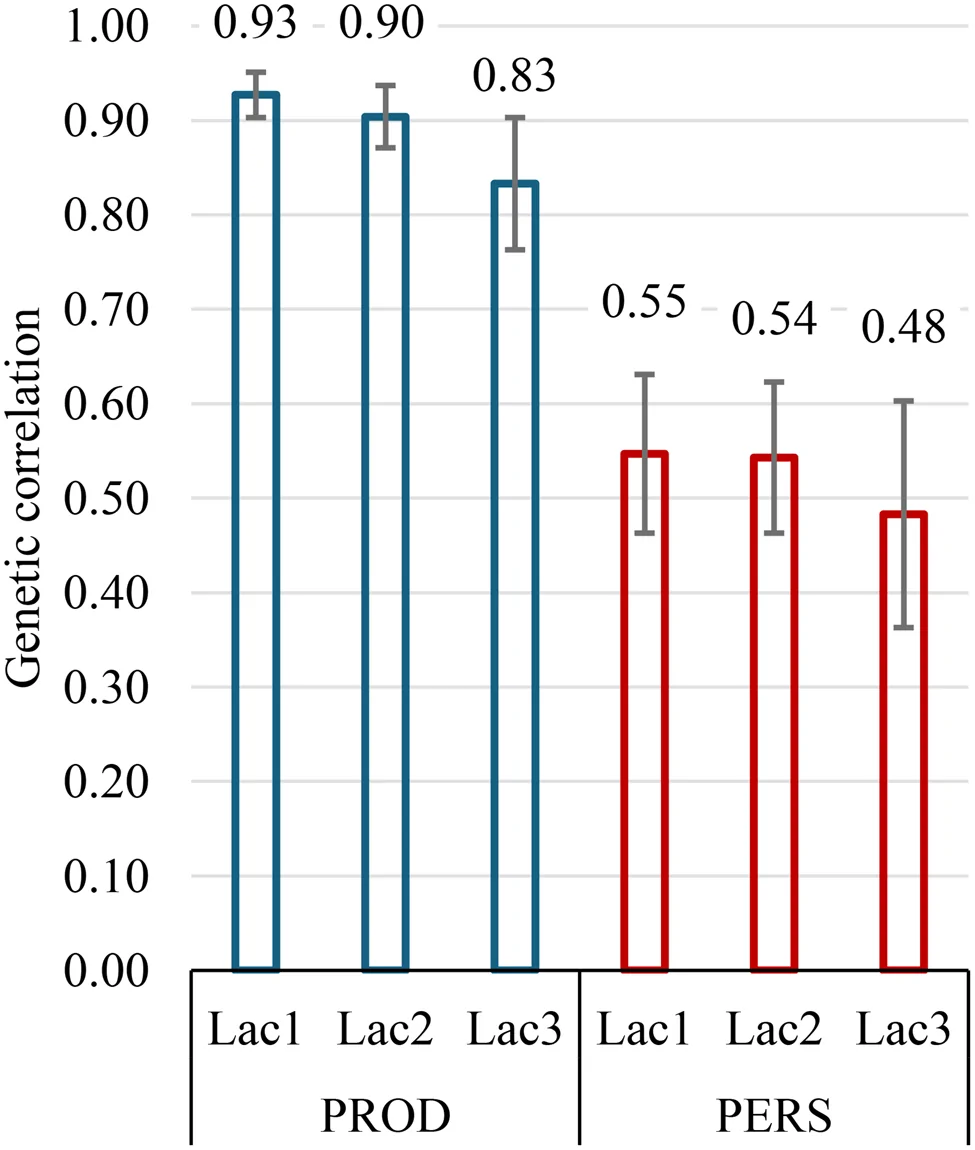
Genetic correlations (standard errors) within lactations between the total mixed ration and pasture production systems for milk production. Laci = lactation i; PROD = production level (blue); PERS = persistency (red)

The moderate genetic correlations (Fig. 9; 0.55, 0.54 and 0.48) obtained for PERS between the production systems indicate that PERS in a TMR system is a different trait from PERS in a PAST system. The genetic correlations are significantly lower than 0.80 [41], showing substantial GxE interaction between TMR and PAST for persistency. These results indicate that different genes are probably at play in the different production systems and that selection in one of the production systems could thus lead to a sub-optimal genetic trend for the trait in the other production system.

Contrary to PERS, the genetic correlations (Milk) between the PROD effects (Fig. 9) were strong (0.93, 0.90 and 0.83), indicating that the same trait is measured in the two production systems. Lac1 and 2 PROD correlations are significantly greater than 0.80 [41] and 1 (*p* < 0.05). Lac3 (95% confidence interval 0.69 to 0.97) may not be significantly different from 0.80 [41]. Corresponding results for PROD and PERS between TMR and PAST in Fat and Protein were similar (See Additional file 9 Fig. S17 and S18).

Comparable TD, multi-lactation RR model studies over different systems are scarce. Herds and countries were grouped into different production systems [42], and additionally [43] argued that different countries can be treated as different production environments. Compared to [42], milk production in SA TMR Holstein herds resembles countries that use more concentrates (United States, Israel and Canada), while SA PAST Holstein herds resemble rotational grazing systems used in New Zealand and Ireland. A significant GxE interaction was indicated by [12] between New Zealand and countries with intensive management systems (Italy and Canada) for total (milk) yield (r_g_ 0.66 and 0.72, respectively) and persistency (r_g_ 0.16 and 0.28, respectively; defined similarly in our study). Amongst all 4 countries (Australia as the 4^th^) used in [12], the genetic correlations for persistency ranged from 0.16 to 0.55. Significant GxE between Luxembourg (high-input system) and Tunisian Holsteins (low- to medium-input systems) was also showed by [34] for 305-day milk yield (r_g_ 0.60) and persistency (r_g_ 0.36; defined as the difference between DIM 80 and 280). Using a TD, single-lactation RR model over low, medium and high production systems, Milk yield genetic correlations varying from 0.04 to 0.54 over different DIM were reported by [26].

Using experimental data, other studies [44–46] also found significant genotype by diet, feeding systems and feeding level interactions, respectively, for milk yield in dairy cattle.

In contrast to the current study, [5] and [6] found negligible differences between conventional (using harvested, stored forage with “free-stall” access) and rotational grazing (low input) systems (r_g_ close to 0.90 or greater). However, these studies [5, 6] used 305-day lactation records and not RR TD models, resulting in an inability to independently distinguish between the genetic persistency vs the genetic (level of) production effects. Due to this reason, [19] also most likely did not find a significant GxE interaction in a preliminary SA Holstein study of milk production in TMR vs PAST (r_g_ = 0.90 ± 0.03).

Genetic correlations between the TMR and PAST (and vice versa) regarding their PROD and PERS components were mostly weak and varied from −0.47 to 0.09, often times not being significantly different from zero (*p* > 0.05). Correspondingly, within-TMR and -PAST genetic correlations between PROD and PERS were also mostly weak and ranged from −0.52 to 0.06, also regularly not being significantly different from zero (*p* < 0.05). Thus, independent genetic improvement of PROD from PERS and vice versa should be easily attainable.

The ideal situation would be to have separate EBV-PERS for the TMR and PAST production systems. TMR herds are predominantly situated in the arid to semi-arid regions, while PAST herds are primarily situated in the higher average annual rainfall regions of SA [21]. PAST herds would benefit from selection to improve persistency as cows typically have to produce over 2.5 seasons in more extensive conditions, giving producers the opportunity to breed more resilient animals in this regard. This might lead to persistency having a different economic value in PAST. TMR herds, typically more intensive, will benefit from genetically improving persistency as there is a favourable genetic response on health traits [14]. Consequently, dissimilar selection indices for PAST vs TMR become apparent. Separate selection indices become more compelling when considering the addition of other economically important traits like age at first calving, where evidence of significant GxE between TMR and PAST in SA Holsteins were shown [19]. Using such a distinct PAST selection index will enable PAST herds to further select for “overall” resilient animals. Traits such as reproduction, although important in both production systems, will probably carry more economic weight in a PAST selection index. In addition, [15] showed the significant effect of persistency on calving interval.

### Genetic correlations between lactations for TMR vs PAST

**Fig. 10:**
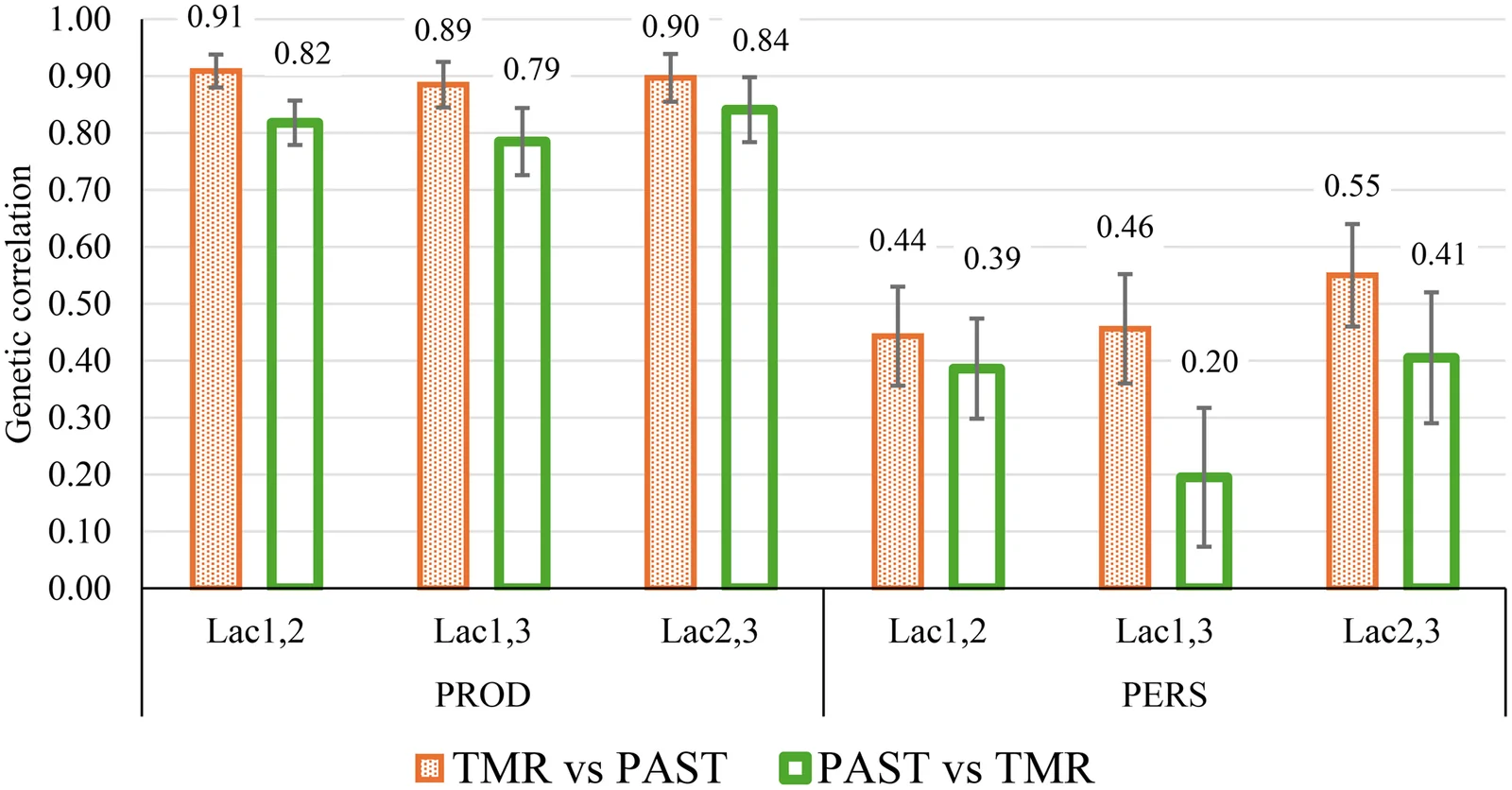
Genetic correlations (standard errors) between lactations for the total mixed ration and pasture production systems for milk production. Laci,j = lactation i,j; PROD = production level; PERS = Persistency; TMR = total mixed ration production system; PAST = pasture production system

Between lactations PERS genetic correlations (Milk) for PAST Lac1 vs TMR later lactations (0.39 and 0.20, respectively; Fig. 10) were weak with vice versa estimates (TMR Lac1 vs PAST later lactations) tending somewhat higher towards moderate (0.44 and 0.46, respectively). This tendency was more pronounced for Fat (0.60 and 0.62, respectively) and Protein (0.62 and 0.61, respectively; Additional file 9 Fig. S19 and S20, respectively). These TMR Lac1 vs PAST later lactations PERS genetic correlations show that TMR Lac1 is a better indicator of PAST later lactations than is the case within PAST (−0.19 to 0.07 for all traits), especially for Fat and Protein (See Additional file 10 Table S1 to S3 for Milk, Fat and Protein, respectively).

### Genetic correlations between TMR and PAST across lactations

**Fig. 11:**
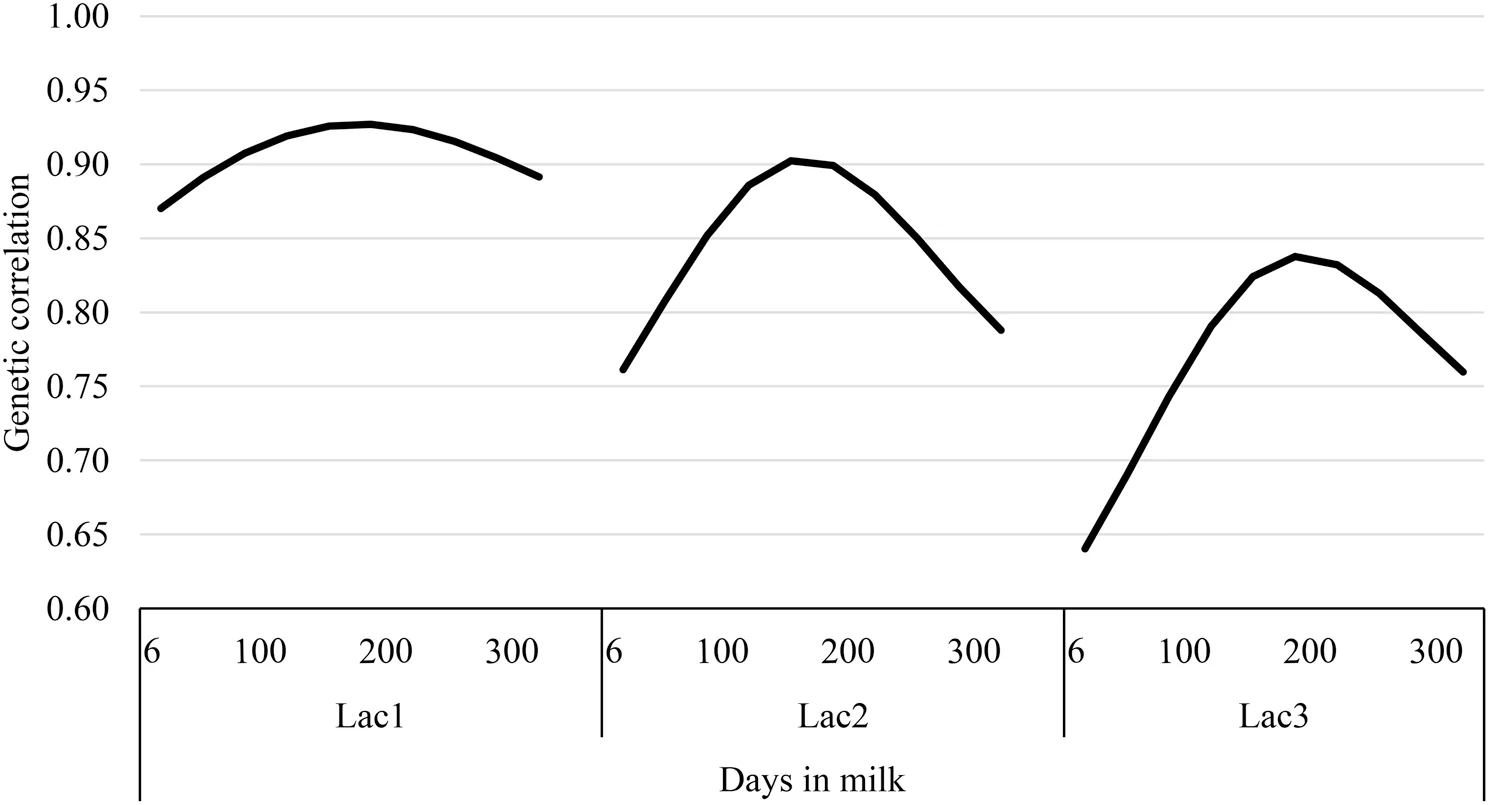
Genetic correlations over days in milk between the total mixed ration and pasture production systems for milk production (TOTAL). Laci = lactation i; TOTAL = total production i.e., production level (PROD) and persistency (PERS)

Genetic correlations (TOTAL; Fig. 11) between TMR and PAST over DIM for Milk were lowest during early and late lactation, especially for Lac2 and Lac3, where it was below 0.80 in some instances. The corresponding genetic correlation curve over DIM for Fat and Protein followed the same tendency (Additional file 11 Fig. S21 and S22, respectively). However, the curves for Fat over the first 3 lactations was almost always above 0.80 at the beginning and end of the lactation. GxE for TOTAL is more pronounced during the beginning and end of lactation, especially for later lactations. The same tendency was found in a GxE study comparing production in warmer climates to other regions [47].

### Sire EBV rank correlations between TMR and PAST

The EBV-PERS percentile rank correlations between TMR and PAST for all sires with at least 5 daughters each in TMR and PAST (shared sires-5; Table 1) were 0.53, 0.50 and 0.45 for Milk Lac1 to Lac3, respectively (not shown). The equivalent correlations (not shown) for Fat Lac1 to Lac3 were 0.52, 0.61 and 0.55, respectively and 0.39, 0.49 and 0.46, respectively for Protein. In this regard, reranking will be significant.

Unlike EBV-PERS, reranking of shared sires-5 for EBV-PROD should be minor with strong percentile rank correlations mostly above 0.92 (range 0.87 to 0.97) for all lactations in Milk, Fat and Protein (not shown).

The EBV-TOTAL percentile rank correlations between TMR and PAST for all shared sires-5 were 0.78, 0.64 and 0.51 for Milk Lac1 to Lac3, respectively (not shown). The corresponding correlations for Fat were 0.72, 0.64 and 0.64, and 0.71, 0.62 and 0.55, respectively for Protein. Accordingly, significant reranking of sires will still take place based on EBV-TOTAL, especially in later lactations and more so than based on EBV-PROD. The corresponding EBV-PERS, -PROD and -TOTAL correlations for all shared sires-20 (not shown) were similar to those of shared sires-5.

Table 4 quantifies the reranking of shared sires for Milk in Lac1, which are in the 70th percentile (i.e., superior) for EBV-PERS, -PROD and TOTAL for PAST (195, 314 and 281 shared sires-5, respectively). A small proportion (24%) of the sires superior in PAST for EBV-PERS, will also be superior for EBV-PERS in TMR, indicating major reranking (Table 4). Correspondingly, the opposite was true for EBV-PROD (95% sires in common) showing minor reranking, while some (moderate) reranking will take place for EBV-TOTAL (83% sires in common). Equivalently, the same trend is apparent for the more influential sires with more recorded daughters (shared sires-20; Table 4). Similarly, shared sires-5 (and −20) in common over PAST and TMR for Fat Lac1, EBV-PERS, -PROD and -TOTAL were 31% (33%), 98% (98%), and 78% (73%), respectively and 15% (18%), 96% (96%), and 80% (78%), respectively for Protein Lac1.

**Table 4:** The number of sires with estimated breeding values (EBVs) in the 70th percentile in pasture (PAST), compared to the number of sires in common (also in the 70th percentile) in the total mixed ration (TMR) production system for milk production in first lactation

**EBVs in 70**^**th**^**percentile**	*n* shared sires-5			*n* shared sires-20		
	PAST	TMR	Sires in common (%)	PAST	TMR	Sires in common (%)
PERS	195	47	24	82	23	28
PROD	314	298	95	115	106	92
TOTAL	281	232	83	100	84	84

*n* = number of; shared sires-5 = sires with a minimum of 5 daughters (with records) in the pasture and total mixed ration production systems each; shared sires-20 = sires with a minimum of 20 daughters (with records) in the pasture and total mixed ration production systems each; PERS = persistency; PROD = production level; TOTAL = total production (PROD and PERS)

In their GxE study of production traits in confined vs grazing herds of primiparous US Holsteins, [7] obtained EBV rank correlations of 0.59, 0.63 and 0.66, also for sires with at least 5 daughters producing in both environments. Across herd management levels for Luxembourg (high-input systems) and Tunisian Holsteins (lower-input systems), [48] showed EBV rank correlations for common sires to be weak, ranging from 0.19 to 0.41 for 305-day milk yield.

Producers should consider both the EBV-PERS and EBV-TOTAL when selecting sires as to fit their selection objectives. A significant influence of EBV-PERS on EBV-TOTAL using a comparable dataset was shown by [49], and can be seen as an example where EBV-PERS is the main selection goal. Sires-5 were grouped on their EBV-PERS (295-days Milk basis) as superior (70th percentile), average (30th to 70th percentile), and inferior (30th percentile) for TMR and PAST, separately. In TMR, the sires that had on average the highest EBV-TOTAL were the superior EBV-PERS sire group, despite having on average the lowest EBV-PROD. In PAST, the average EBV-PROD of the superior, average and inferior sire groups were close to each other (much more so than in TMR), with the superior EBV-PERS sire group having the highest EBV-TOTAL, as with TMR. By considering both these EBVs, producers can select sires that will have daughters that produce a certain (optimal) total production volume over the lactation, by ways of either having an above average EBV-PERS (favourable genetic merit for maintained production), or EBV-PROD (favourable genetic merit for level of production), or both. Hence, because of the presence of GxE for PERS, sires should have distinct EBVs for traits where GxE is present, so that producers can select bulls that will breed cows to fit their production system (TMR or PAST). Genetic links between herds through sires used (shared) amongst TMR and PAST should be initiated and kept intact as to ascertain the genetic merit for especially persistency in TMR vs PAST. This will also aid producers to breed specialised breeding lines (genetic plasticity) where sires breed favourably in a chosen system only, or more resilient breeding lines where sires breed relatively favourably in both systems with regards to PERS.

Achieving favourable selection response for set breeding goals, analysing production in TMR and PAST together, as separate traits in a multi-trait genetic evaluation is key, as it will lead to more accurate EBVs as the extra information from the genetic covariance matrix is utilized. Because the genetic correlations between especially TMR vs PAST are significantly greater than 0, sires’ daughters performance in one production system will provide valuable statistical information about their genetic merit in the other production system, and vice versa. This is especially true for PERS in TMR vs PAST for Fat and Protein. Separate analyses of production systems will not account for the fact that biological performance in one system provides a baseline genetic merit for the other. Increased accuracy as described is particularly important where sire data is unbalanced between TMR and PAST, which is often the case. Gains in EBV accuracy (reliability) for predicting sire performance in diverse agricultural conditions like SA, will certainly outweigh higher multi-trait model computational resource demands. Lastly, for the use of a total economic index accounting for the national herd (which extend over both TMR and PAST), the genetic covariance between the systems is needed to appropriately weight the selection index.

## Conclusions

Genetic correlations show a significant genotype by environment interaction for the genetic persistency effect between the total mixed ration and pasture production systems in the South African Holstein population. This result is for all lactations in all traits, and all genetic correlations were significantly different from 1, and almost all from 0.80. Sire reranking results show significant reranking of sires between the two production systems for EBV-PERS, but also for EBV-TOTAL. The effect of EBV-PROD (minor GxE) to compensate for the major GxE in EBV-PERS do not seem large enough to significantly lessen the reranking of sires on EBV-TOTAL. To accomplish faster genetic gain through more accurate genetic predictions, each participating herd must record their production system used. Moreover, the implication for genetic evaluations is the treatment of production traits in the two production systems as genetically different traits, utilizing an applicable random regression model that can effectively dissociate genetic production level from genetic persistency. Results justify the publication of separate estimate breeding values for persistency for each production system.

## Electronic supplementary material

Below is the link to the electronic supplementary material.

Supplementary Material 1

Supplementary Material 2

Supplementary Material 3

Supplementary Material 4

Supplementary Material 5

Supplementary Material 6

Supplementary Material 7

Supplementary Material 8

Supplementary Material 9

Supplementary Material 10

Supplementary Material 11

## Data Availability

The data that support the findings of this study are available from the Holstein Cattle Breeders’ Society of South Africa, but restrictions apply. The data were used under license for the current study, and so are not publicly available. Data are however available from the authors upon reasonable request and with permission of the Holstein Cattle Breeders’ Society of South Africa.
