# Supplementary material for: Genotype by environment interaction under two production systems utilizing a random regression model for milk production traits in multiparous South African Holstein dairy cattle
Source: Genet Sel Evol. 2026 Oct 30;58:78. doi: 10.1186/s12711-026-01098-x (PMC13723095; doi:10.1186/s12711-026-01098-x)
Supplement: Supplementary file 1 [file 12711_2026_1098_MOESM10_ESM.pdf]

Additional file 10:

Genotype by environment interaction under two production systems utilizing a random regression model for milk production traits in multiparous South African Holstein dairy cattle

Van Niekerk et al. (2026) Genetics Selection Evolution

Table S1 Genetic correlations (Milk) within and between the TMR and PAST production systems

			TMR						PAST					
			Lac1		Lac2		Lac3		Lac1		Lac2		Lac3	
			PROD	PERS	PROD	PERS	PROD	PERS	PROD	PERS	PROD	PERS	PROD	PERS
TMR	Lac1	PROD	1.00											
		PERS	-0.40_{5}	1.00										
	Lac2	PROD	0.89_{2}	-0.51_{5}	1.00									
		PERS	-0.06_{5}	0.18_{6}	-0.20_{5}	1.00								
	Lac3	PROD	0.90_{4}	-0.39_{7}	0.96_{3}	-0.09_{7}	1.00							
		PERS	-0.15_{7}	0.14_{9}	-0.25_{8}	0.88_{5}	-0.19_{10}	1.00						
PAST	Lac1	PROD	**0.93_{2}**	-0.39_{6}^{e}	0.82_{4}^{b}	-0.05_{6}^{e}	0.79_{6}^{b}	-0.20_{9}^{e}	1.00					
		PERS	-0.01_{7}^{e}	**0.55_{8}**	-0.04_{8}^{e}	0.39_{9}^{d}	0.18_{10}^{e}	0.20_{12}^{d}	-0.01_{6}	1.00				
	Lac2	PROD	0.91_{3}^{a}	-0.50_{6}^{e}	**0.90_{3}**	-0.16_{7}^{e}	0.84_{6}^{b}	-0.28_{9}^{e}	0.95_{2}	-0.15_{7}	1.00			
		PERS	-0.07_{7}^{e}	0.44_{9}^{c}	-0.04_{8}^{e}	**0.54_{8}^{b}**	-0.02_{10}^{e}	0.41_{12}^{d}	0.07_{6}	0.28_{8}	-0.02_{7}	1.00		
	Lac3	PROD	0.89_{4}^{a}	-0.46_{8}^{e}	0.90_{4}^{a}	-0.12_{8}^{e}	**0.83_{7}**	-0.25_{10}^{e}	0.92_{3}	-0.17_{8}	0.98_{2}	0.01_{8}	1.00	
		PERS	-0.02_{8}^{e}	0.46_{10}^{c}	-0.10_{9}^{e}	0.55_{9}^{c}	-0.08_{11}^{e}	**0.48_{12}**	-0.03_{7}	0.30_{9}	-0.09_{7}	0.96_{4}	-0.09_{8}	1.00

Milk = Milk production; TMR = Total mixed production system; PAST = Pasture production system; Laci = Lactation i; PROD = Production level; PERS = Persistency; Standard errors: Subscript i * 10^{-2}; **Bold** = Within lactation genetic correlations, PROD and PERS effects between TMR and PAST**;** Superscripts: a = Between lactations genetic correlations of PROD effects, TMR vs PAST; b = Between lactations genetic correlations of PROD effects, PAST vs TMR; c = Between lactations genetic correlations of PERS effects, TMR vs PAST; d = Between lactations genetic correlations of PERS effects, PAST vs TMR; e = Within and between lactation genetic correlations, PROD vs PERS effects (and vice versa) between TMR and PAST.

Additional file 10:

Genotype by environment interaction under two production systems utilizing a random regression model for milk production traits in multiparous South African Holstein dairy cattle

Van Niekerk et al. (2026) Genetics Selection Evolution

Table S2 Genetic correlations (Fat) within and between the TMR and PAST production systems

			**TMR**						**PAST**					
			**Lac1**		**Lac2**		**Lac3**		**Lac1**		**Lac2**		**Lac3**	
			**PROD**	**PERS**	**PROD**	**PERS**	**PROD**	**PERS**	**PROD**	**PERS**	**PROD**	**PERS**	**PROD**	**PERS**
**TMR**	**Lac1**	**PROD**	1.00											
		**PERS**	-0.26_{6}	1.00										
	**Lac2**	**PROD**	0.86_{2}	-0.38_{7}	1.00									
		**PERS**	-0.02_{6}	0.18_{8}	-0.10_{7}	1.00								
	**Lac3**	**PROD**	0.89_{4}	-0.30_{9}	0.97_{3}	-0.03_{8}	1.00							
		**PERS**	0.01_{8}	0.02_{11}	0.03_{9}	0.93_{6}	0.06_{11}	1.00						
**PAST**	**Lac1**	**PROD**	$\mathbf{0.89_{3}}$	-0.27_{8}^{e}	0.84_{4}^{b}	0.08_{8}^{e}	0.90_{5}^{b}	0.13_{10}^{e}	1.00					
		**PERS**	0.09_{9}^{e}	$\mathbf{0.55_{11}}$	0.10_{10}^{e}	0.42_{11}^{d}	0.22_{11}^{e}	0.28_{15}^{d}	0.01_{8}	1.00				
	**Lac2**	**PROD**	0.86_{4}^{a}	-0.36_{8}^{e}	$\mathbf{0.93_{4}}$	-0.05_{8}^{e}	0.94_{5}^{b}	0.05_{11}^{e}	0.96_{2}	-0.05_{9}	1.00			
		**PERS**	-0.09_{9}^{e}	0.60_{10}^{c}	-0.23_{9}^{e}	$\mathbf{0.60_{10}}$	-0.22_{11}^{e}	0.60_{13}^{d}	-0.05_{8}	0.22_{11}	-0.20_{8}	1.00		
	**Lac3**	**PROD**	0.86_{5}^{a}	-0.32_{10}^{e}	0.93_{5}^{a}	-0.02_{10}^{e}	$\mathbf{0.92_{7}}$	0.02_{12}^{e}	0.91_{4}	0.02_{10}	0.96_{3}	-0.19_{9}	1.00	
		**PERS**	-0.17_{10}^{e}	0.62_{12}^{c}	-0.26_{11}^{e}	0.58_{12}^{c}	-0.25_{13}^{e}	$\mathbf{0.60_{14}}$	-0.19_{9}	0.38_{12}	-0.29_{10}	0.94_{6}	-0.31_{11}	1.00

Fat = Fat production; TMR = Total mixed production system; PAST = Pasture production system; Laci = Lactation i; PROD = Production level; PERS = Persistency; Standard errors: Subscript i * 10^{-2} ; **Bold** = Within lactation genetic correlations, PROD and PERS effects between TMR and PAST**;** Superscripts: a = Between lactations genetic correlations of PROD effects, TMR vs PAST; b = Between lactations genetic correlations of PROD effects, PAST vs TMR; c = Between lactations genetic correlations of PERS effects, TMR vs PAST; d = Between lactations genetic correlations of PERS effects, PAST vs TMR; e = Within and between lactation genetic correlations, PROD vs PERS effects (and vice versa) between TMR and PAST.

Additional file 10:

Genotype by environment interaction under two production systems utilizing a random regression model for milk production traits in multiparous South African Holstein dairy cattle

Van Niekerk et al. (2026) Genetics Selection Evolution

Table S3 Genetic correlations (Protein) within and between the TMR and PAST production systems

			TMR						PAST					
			Lac1		Lac2		Lac3		Lac1		Lac2		Lac3	
			PROD	PERS	PROD	PERS	PROD	PERS	PROD	PERS	PROD	PERS	PROD	PERS
TMR	Lac1	PROD	1.00											
		PERS	-0.52_{4}	1.00										
	Lac2	PROD	0.88_{2}	-0.54_{5}	1.00									
		PERS	-0.09_{5}	0.16_{7}	-0.38_{5}	1.00								
	Lac3	PROD	0.88_{4}	-0.48_{7}	0.98_{3}	-0.29_{7}	1.00							
		PERS	-0.16_{7}	0.18_{9}	-0.39_{7}	0.83_{6}	-0.31_{9}	1.00						
PAST	Lac1	PROD	$\mathbf{0.90_{3}}$	-0.47_{6}^{e}	0.81_{4}^{b}	-0.08_{7}^{e}	0.75_{6}^{b}	-0.21_{9}^{e}	1.00					
		PERS	0.03_{8}^{e}	$\mathbf{0.40_{10}}$	-0.03_{9}^{e}	0.33_{10}^{d}	0.09_{11}^{e}	0.14_{13}^{d}	-0.03_{7}	1.00				
	Lac2	PROD	0.88_{3}^{a}	-0.57_{7}^{e}	$\mathbf{0.90_{3}}$	-0.22_{7}^{e}	0.85_{6}^{b}	-0.27_{9}^{e}	0.95_{2}	-0.14_{8}	1.00			
		PERS	-0.03_{8}^{e}	0.62_{9}^{c}	-0.21_{9}^{e}	$\mathbf{0.48_{10}}$	-0.21_{11}^{e}	0.36_{13}^{d}	0.01_{7}	0.28_{10}	-0.15_{8}	1.00		
	Lac3	PROD	0.87_{5}^{a}	-0.54_{8}^{e}	0.88_{5}^{a}	-0.13_{9}^{e}	$\mathbf{0.83_{7}}$	-0.22_{10}^{e}	0.95_{3}	-0.17_{9}	0.99_{2}	-0.09_{9}	1.00	
		PERS	-0.14_{9}^{e}	0.61_{10}^{c}	-0.29_{10}	0.49_{11}^{c}	-0.27_{12}^{e}	$\mathbf{0.51_{13}}$	-0.13_{8}	0.34_{11}	-0.25_{9}	0.93_{6}	-0.22_{9}	1.00

Protein = Protein production; TMR = Total mixed production system; PAST = Pasture production system; Laci = Lactation i; PROD = Production level; PERS = Persistency; Standard errors: Subscript i * 10^{-2} ; **Bold** = Within lactation genetic correlations, PROD and PERS effects between TMR and PAST**;** Superscripts: a = Between lactations genetic correlations of PROD effects, TMR vs PAST; b = Between lactations genetic correlations of PROD effects, PAST vs TMR; c = Between lactations genetic correlations of PERS effects, TMR vs PAST; d = Between lactations genetic correlations of PERS effects, PAST vs TMR; e = Within and between lactation genetic correlations, PROD vs PERS effects (and vice versa) between TMR and PAST.
