# Supplementary material for: Genotype by environment interaction under two production systems utilizing a random regression model for milk production traits in multiparous South African Holstein dairy cattle
Source: Genet Sel Evol. 2026 Oct 30;58:78. doi: 10.1186/s12711-026-01098-x (PMC13723095; doi:10.1186/s12711-026-01098-x)
Supplement: Supplementary file 2 [file 12711_2026_1098_MOESM11_ESM.pdf]

**Additional file 11:**

Genotype by environment interaction under two production systems utilizing a random regression model for milk production traits in multiparous South African Holstein dairy cattle

Van Niekerk et al. (2026) Genetics Selection Evolution

**Figure S21 and 22** Genetic correlations over days in milk between the total mixed ration and pasture production systems for Fat and Protein production (TOTAL)

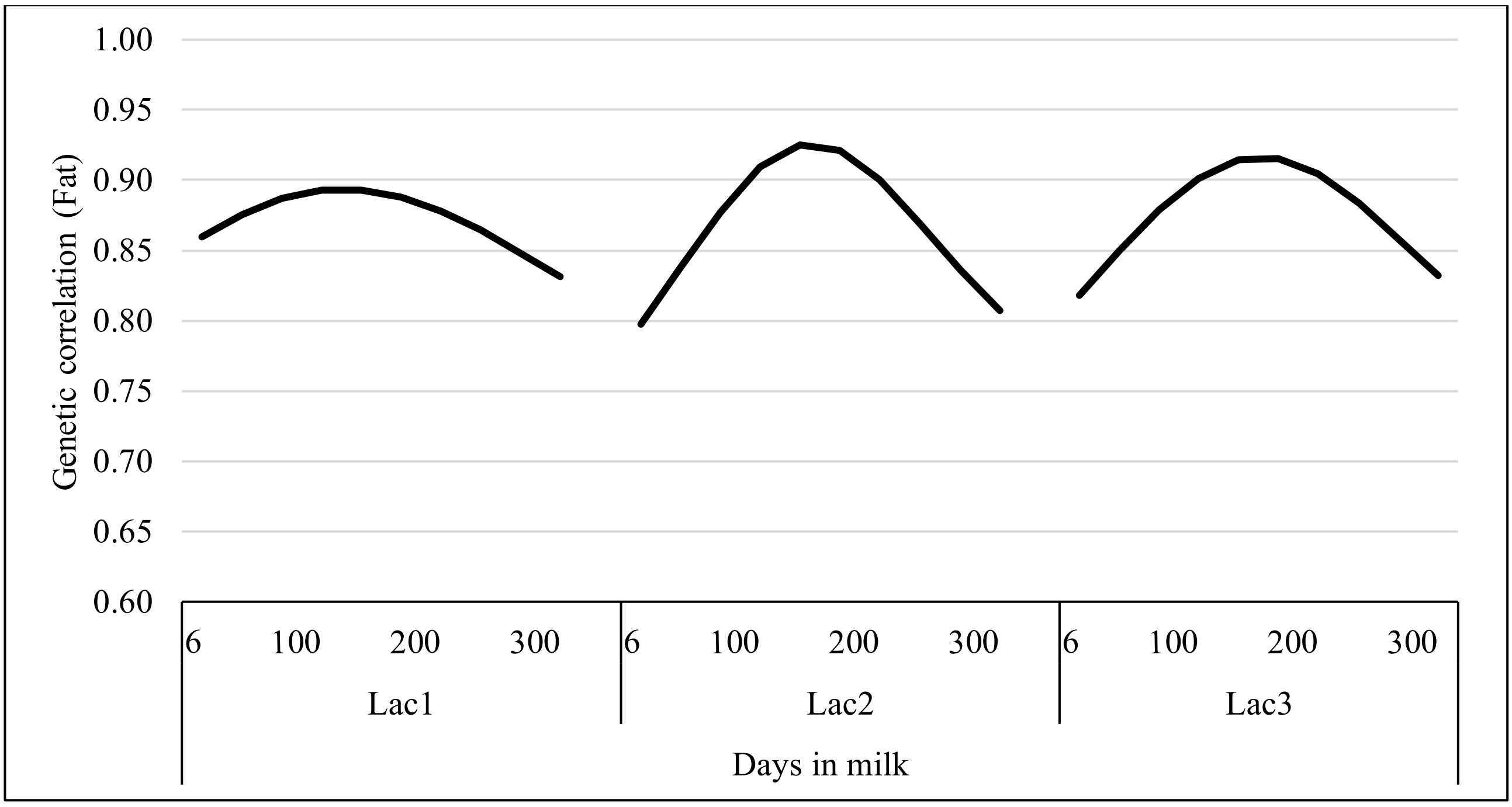


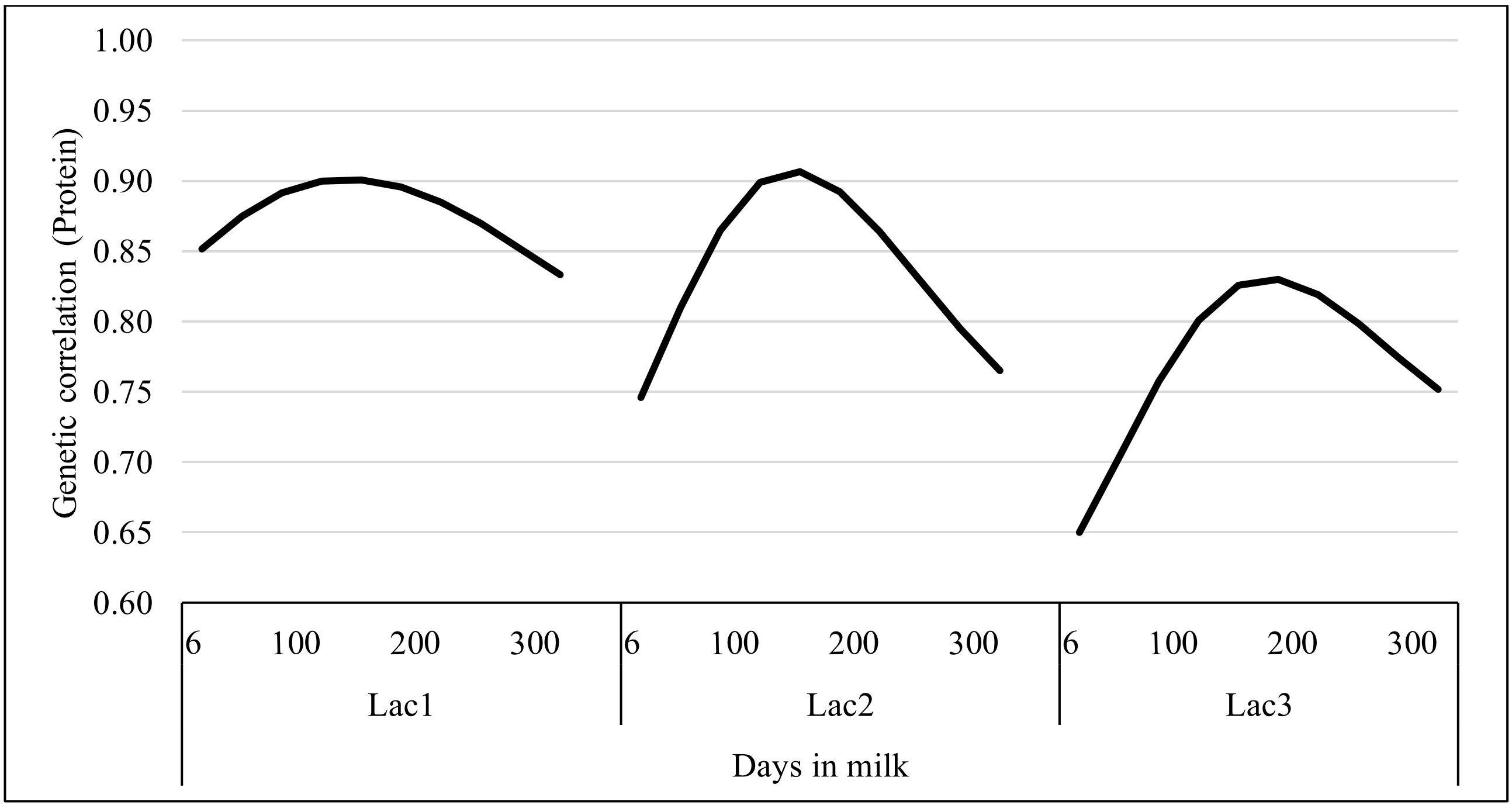


Laci = Lactation i; TOTAL = total production i.e., production level (PROD) and persistency (PERS)
