# Supplementary material for: Genotype by environment interaction under two production systems utilizing a random regression model for milk production traits in multiparous South African Holstein dairy cattle
Source: Genet Sel Evol. 2026 Oct 30;58:78. doi: 10.1186/s12711-026-01098-x (PMC13723095; doi:10.1186/s12711-026-01098-x)
Supplement: Supplementary file 3 [file 12711_2026_1098_MOESM1_ESM.pdf]

Additional file 1:

Genotype by environment interaction under two production systems utilizing a random regression model for milk production traits in multiparous South African Holstein dairy cattle

Van Niekerk et al. (2026) Genetics Selection Evolution

Figures S1, S2 and S3 Genetic, permanent environmental and residual variances for Fat production over days in milk in Lactation 1, 2 and 3

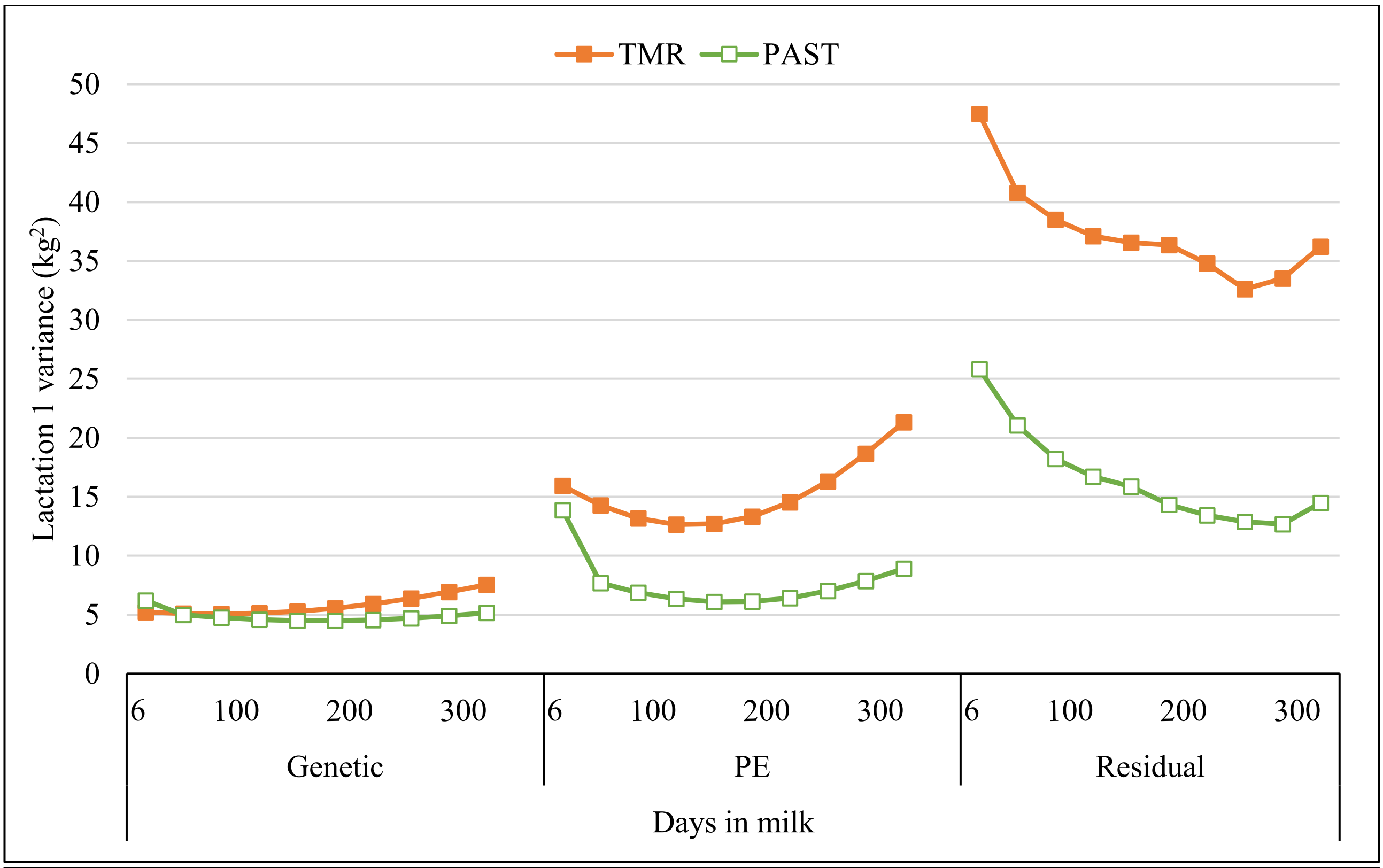

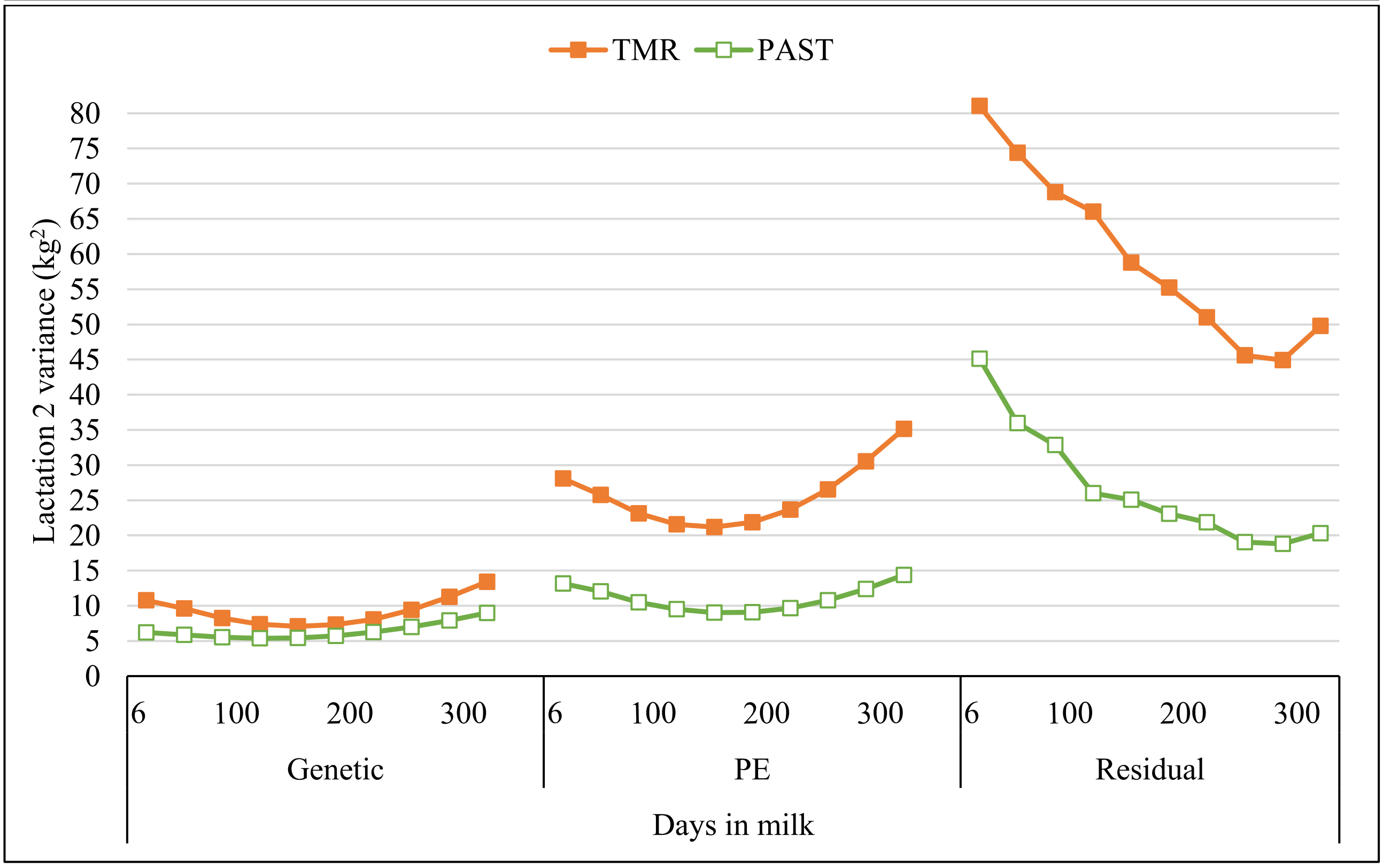

Additional file 1:

Genotype by environment interaction under two production systems utilizing a random regression model for milk production traits in multiparous South African Holstein dairy cattle

Van Niekerk et al. (2026) Genetics Selection Evolution

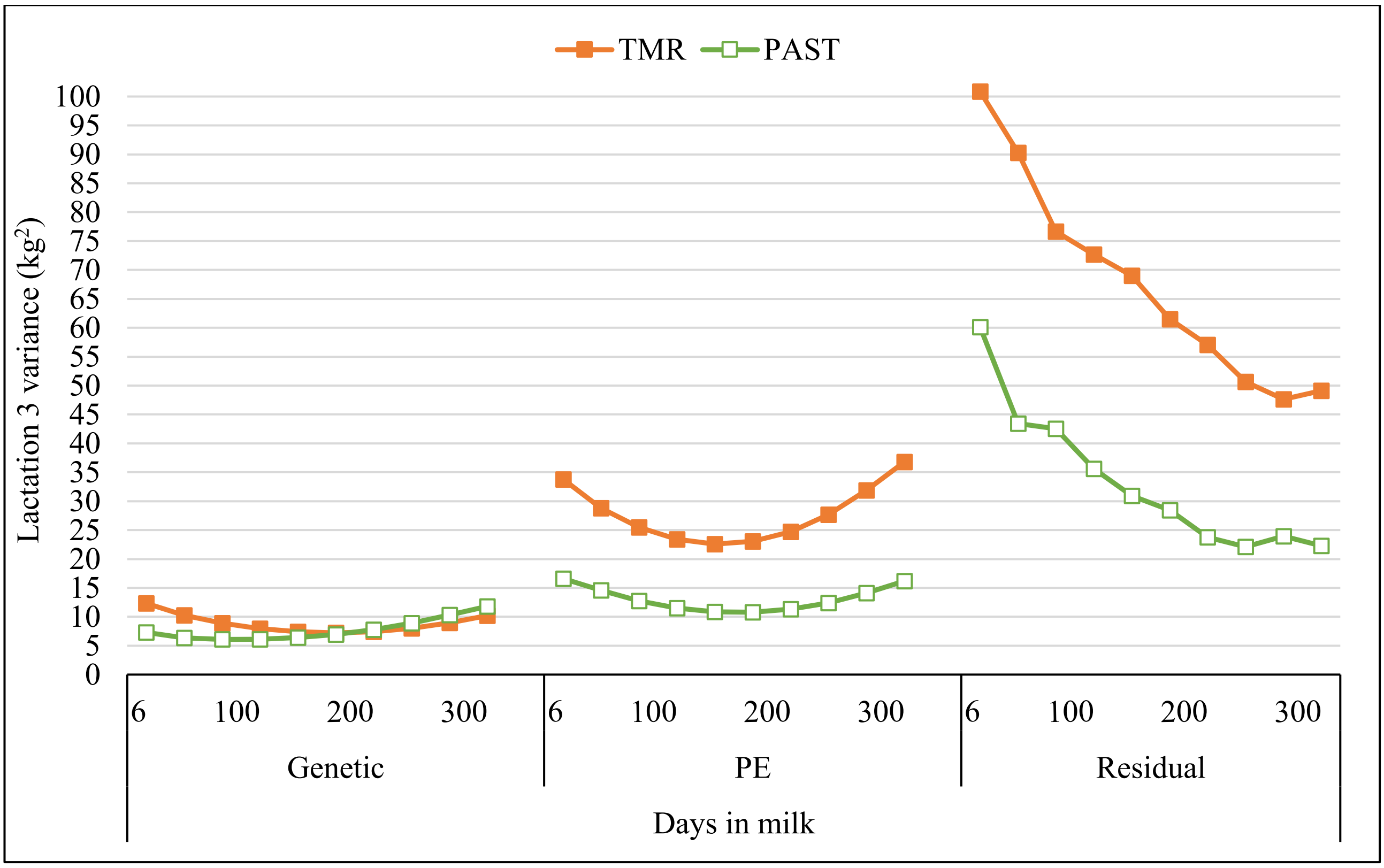

PE = Permanent environmental; TMR = Total Mixed Ration production system; PAST = Pasture production system
