# Supplementary material for: Genotype by environment interaction under two production systems utilizing a random regression model for milk production traits in multiparous South African Holstein dairy cattle
Source: Genet Sel Evol. 2026 Oct 30;58:78. doi: 10.1186/s12711-026-01098-x (PMC13723095; doi:10.1186/s12711-026-01098-x)
Supplement: Supplementary file 5 [file 12711_2026_1098_MOESM3_ESM.pdf]

Additional file 3:

Genotype by environment interaction under two production systems utilizing a random regression model for milk production traits in multiparous South African Holstein dairy cattle

Van Niekerk et al. (2026) Genetics Selection Evolution

Figure S7 Heritability estimates over days in milk for TOTAL Fat production

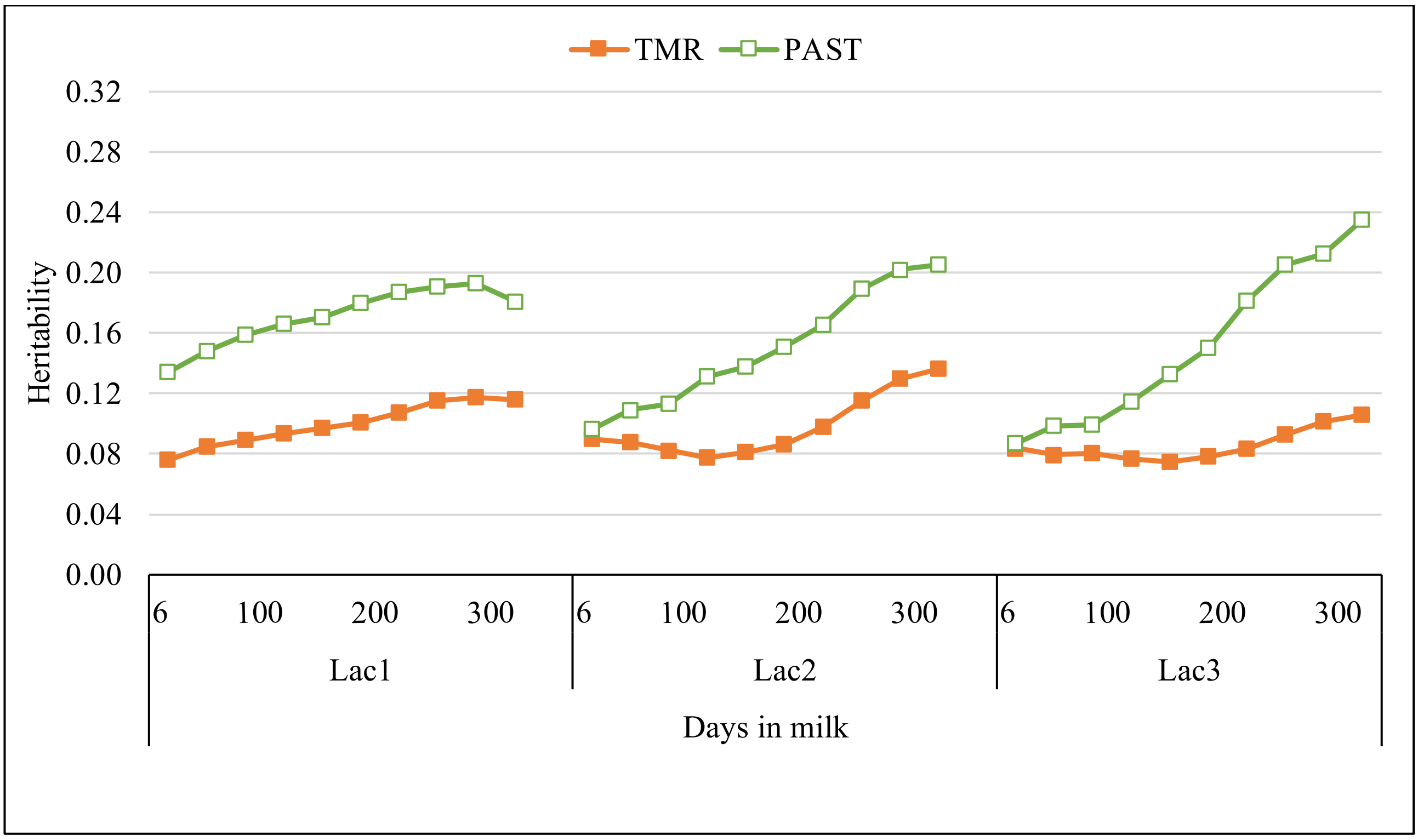

TMR = Total Mixed Ration production system; PAST = Pasture production system; Laci = Lactation i; TOTAL = Total production i.e., production level (PROD) and persistency (PERS); Standard errors for estimates ranged around 0.01, and from 0.01 to 0.03 for TMR and PAST, respectively.
