# Supplementary material for: Genotype by environment interaction under two production systems utilizing a random regression model for milk production traits in multiparous South African Holstein dairy cattle
Source: Genet Sel Evol. 2026 Oct 30;58:78. doi: 10.1186/s12711-026-01098-x (PMC13723095; doi:10.1186/s12711-026-01098-x)
Supplement: Supplementary file 7 [file 12711_2026_1098_MOESM5_ESM.pdf]

Additional file 5:

Genotype by environment interaction under two production systems utilizing a random regression model for milk production traits in multiparous South African Holstein dairy cattle

Van Niekerk et al. (2026) Genetics Selection Evolution

Figures S9, S10 and S11 Permanent environmental and residual variance ratios over days in milk for Fat production in Lactation 1, 2 and 3

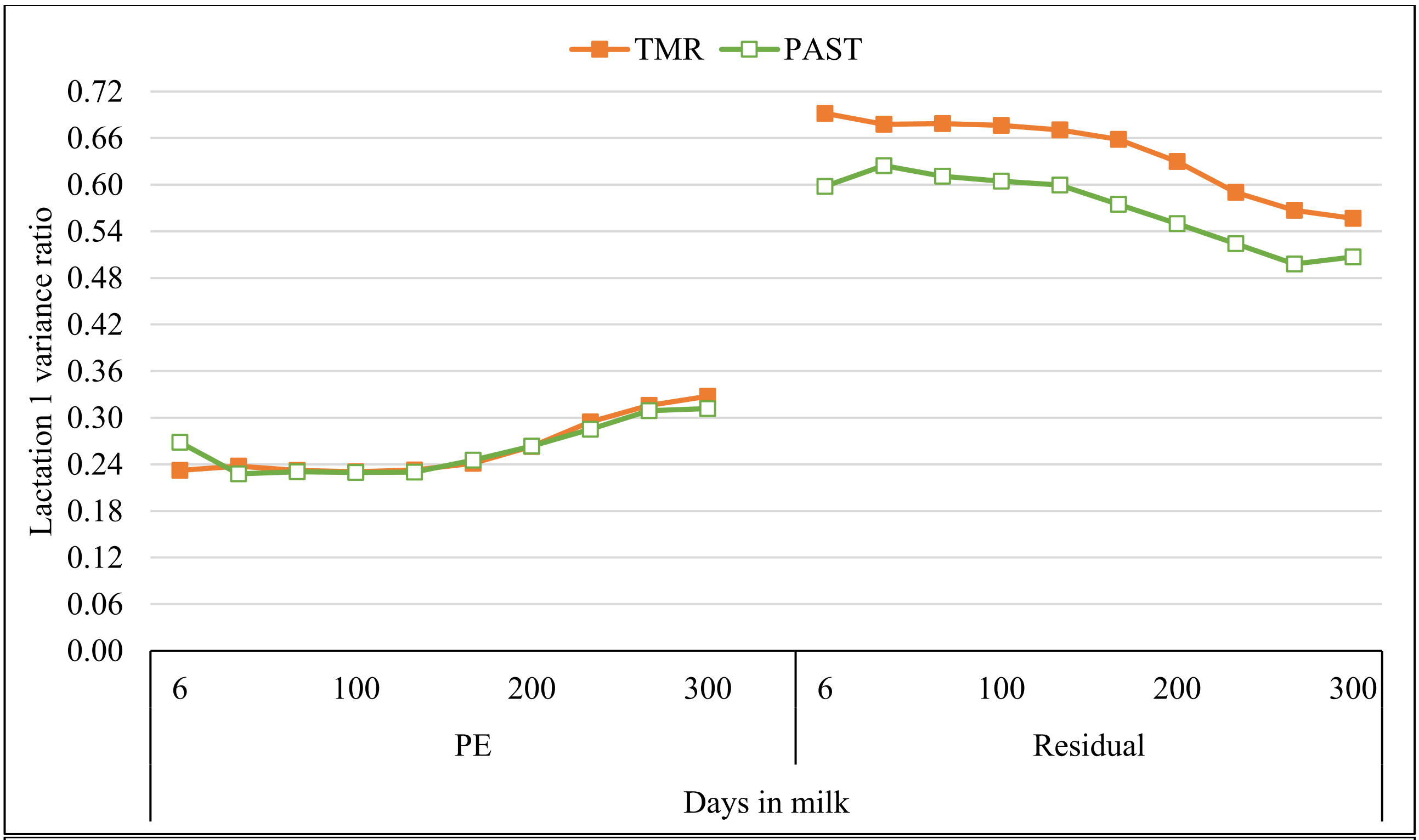

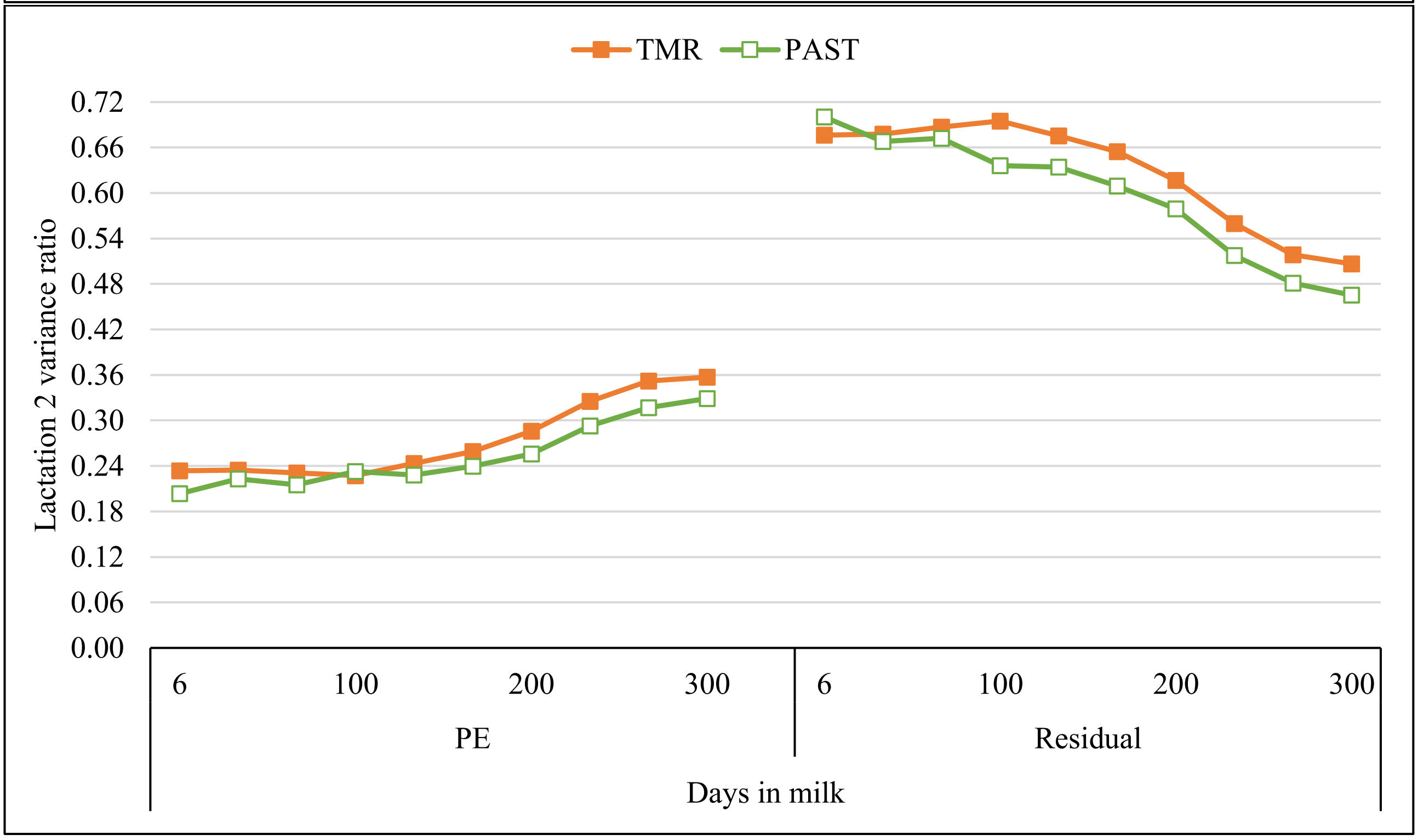

Additional file 5:

Genotype by environment interaction under two production systems utilizing a random regression model for milk production traits in multiparous South African Holstein dairy cattle

Van Niekerk et al. (2026) Genetics Selection Evolution

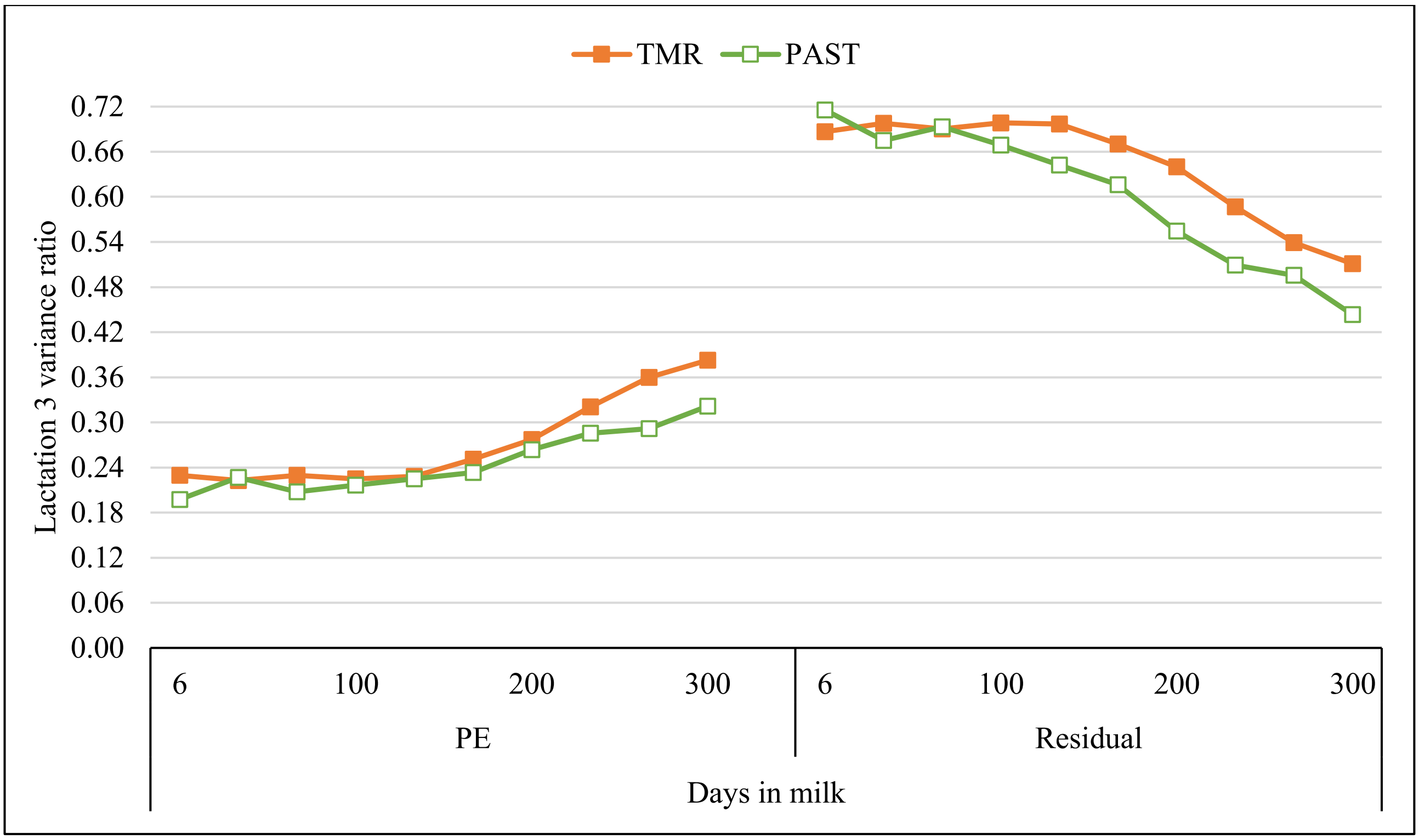

TMR = Total Mixed Ration production system; PAST = Pasture production system; PE = permanent environmental; Standard errors for the permanent environmental ratio estimates ranged from 0.01 to 0.02, and 0.01 to 0.03 for TMR and PAST, respectively.
