# Supplementary material for: Genotype by environment interaction under two production systems utilizing a random regression model for milk production traits in multiparous South African Holstein dairy cattle
Source: Genet Sel Evol. 2026 Oct 30;58:78. doi: 10.1186/s12711-026-01098-x (PMC13723095; doi:10.1186/s12711-026-01098-x)
Supplement: Supplementary file 10 [file 12711_2026_1098_MOESM8_ESM.pdf]

Additional file 8:

Genotype by environment interaction under two production systems utilizing a random regression model for milk production traits in multiparous South African Holstein dairy cattle

Van Niekerk et al. (2026) Genetics Selection Evolution

Figure S16 Heritability estimates in the first 3 lactations for production level and persistency of Protein production

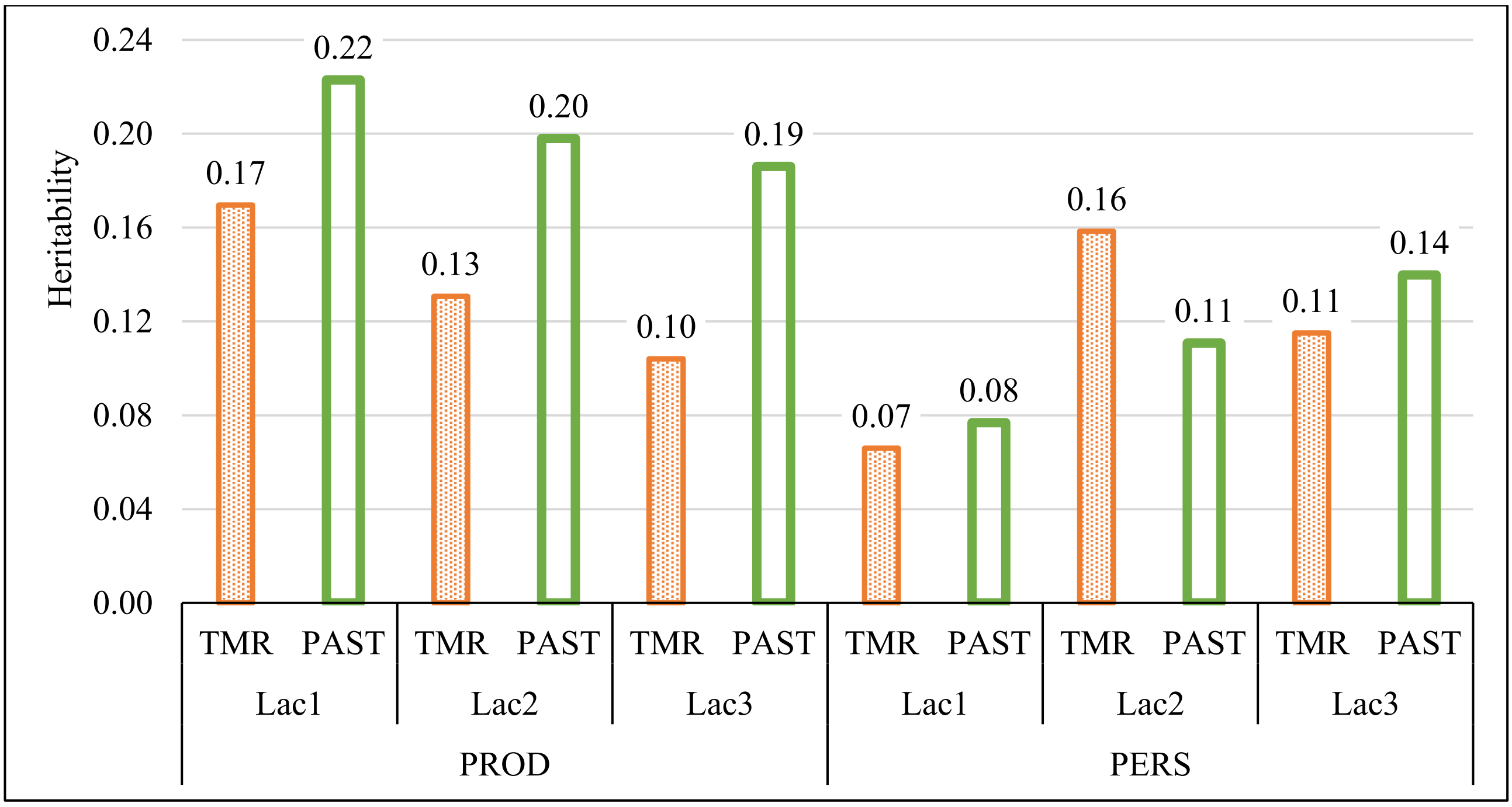

PROD = Production level; PERS = Persistency; TMR = Total mixed ration production system; PAST = Pasture production system; Laci = Lactation i.
