# Supplementary material for: Genotype by environment interaction under two production systems utilizing a random regression model for milk production traits in multiparous South African Holstein dairy cattle
Source: Genet Sel Evol. 2026 Oct 30;58:78. doi: 10.1186/s12711-026-01098-x (PMC13723095; doi:10.1186/s12711-026-01098-x)
Supplement: Supplementary file 11 [file 12711_2026_1098_MOESM9_ESM.pdf]

Additional file 9:

Genotype by environment interaction under two production systems utilizing a random regression model for milk production traits in multiparous South African Holstein dairy cattle

Van Niekerk et al. (2026) Genetics Selection Evolution

Figure S17 Genetic correlations (standard errors) within lactations between the total mixed ration and pasture production systems for Fat production

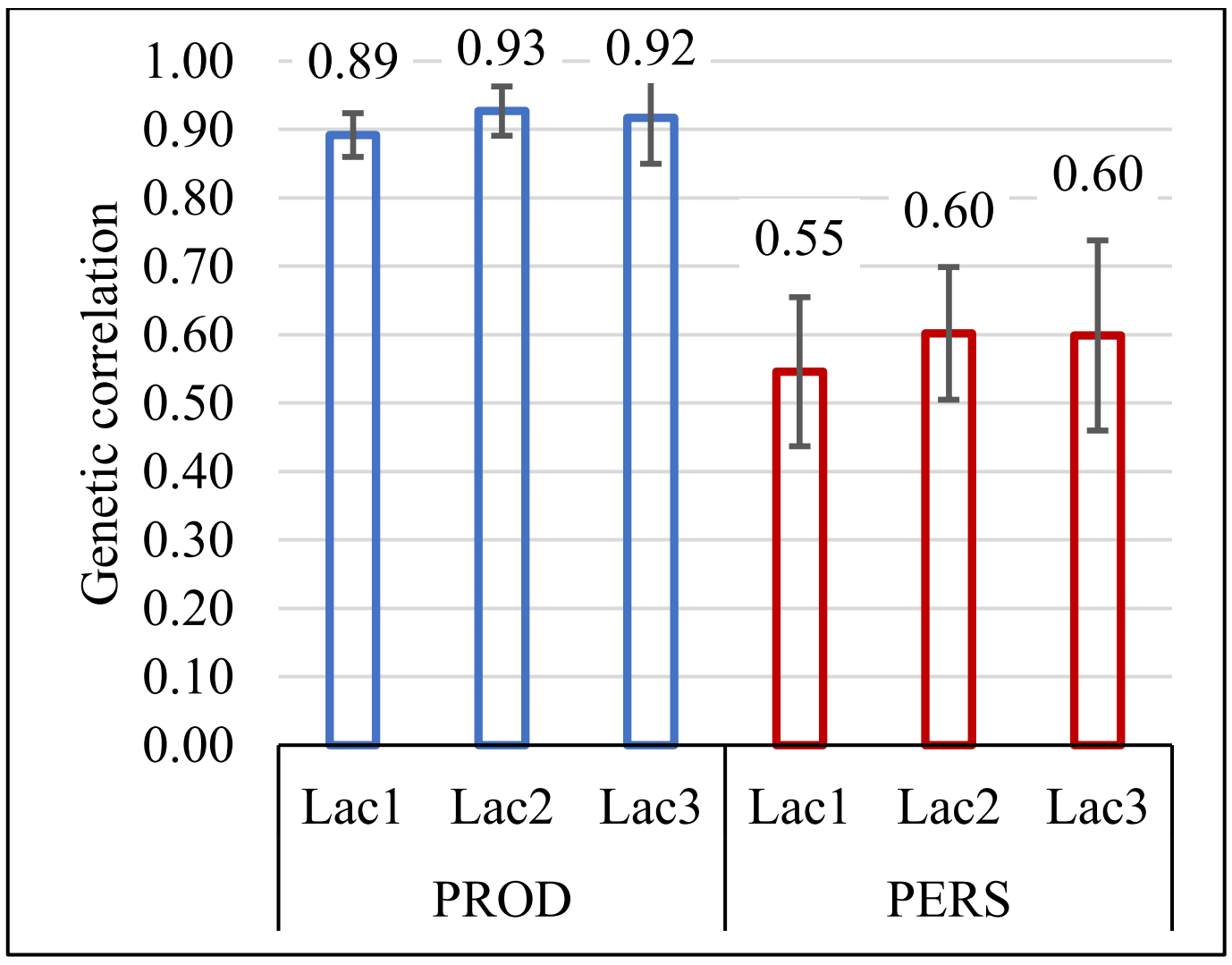

Laci = Lactation i; PROD = Production level (blue); PERS = Persistency (red)

Figure S18 Genetic correlations (standard errors) within lactations between the total mixed ration and pasture production systems for Protein production

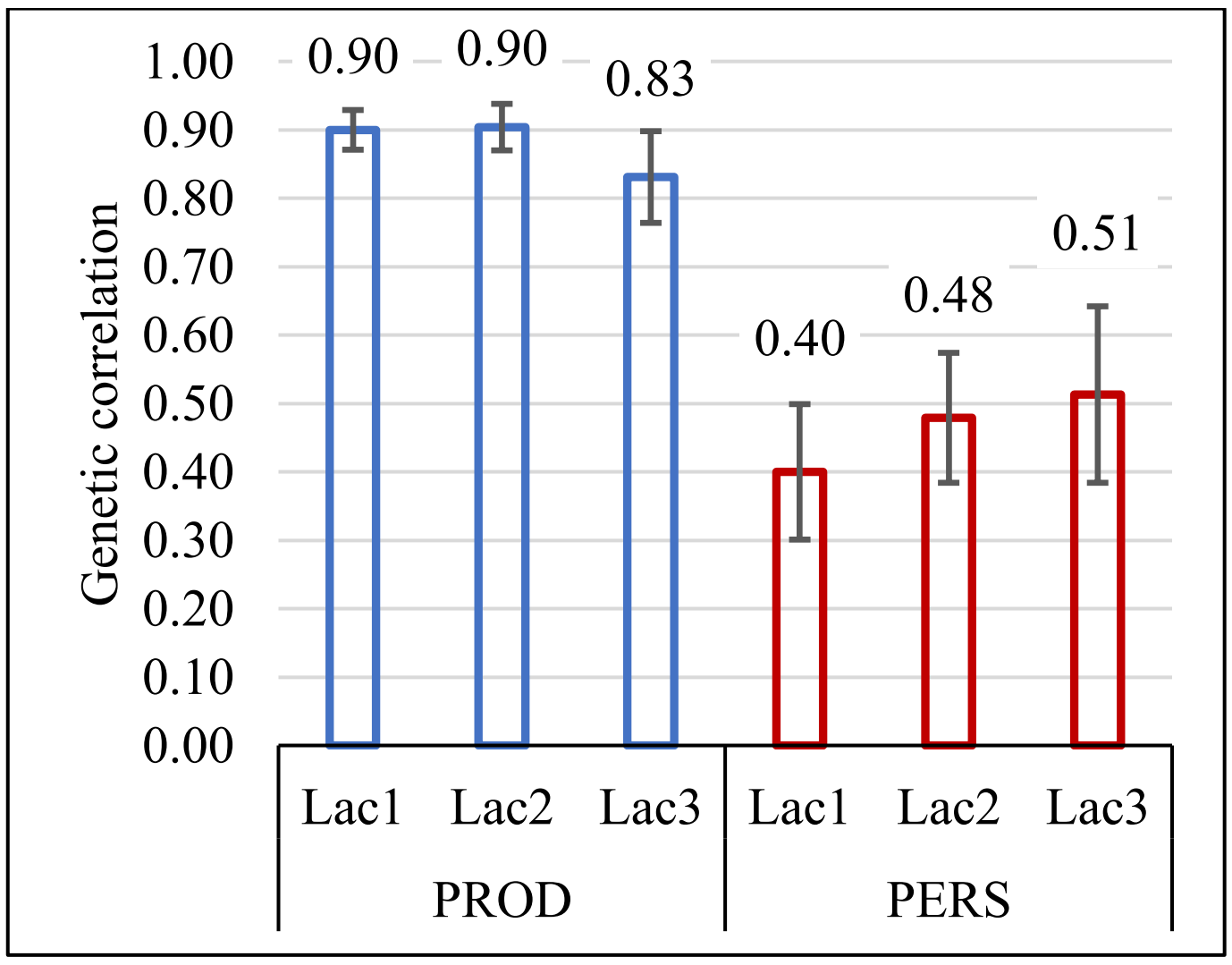

Laci = Lactation i; PROD = Production level (blue); PERS = Persistency (red)

Additional file 9:

Genotype by environment interaction under two production systems utilizing a random regression model for milk production traits in multiparous South African Holstein dairy cattle

Van Niekerk et al. (2026) Genetics Selection Evolution

Figure S19 Genetic correlations (standard errors) between lactations for the total mixed ration vs the pasture production system for Fat production

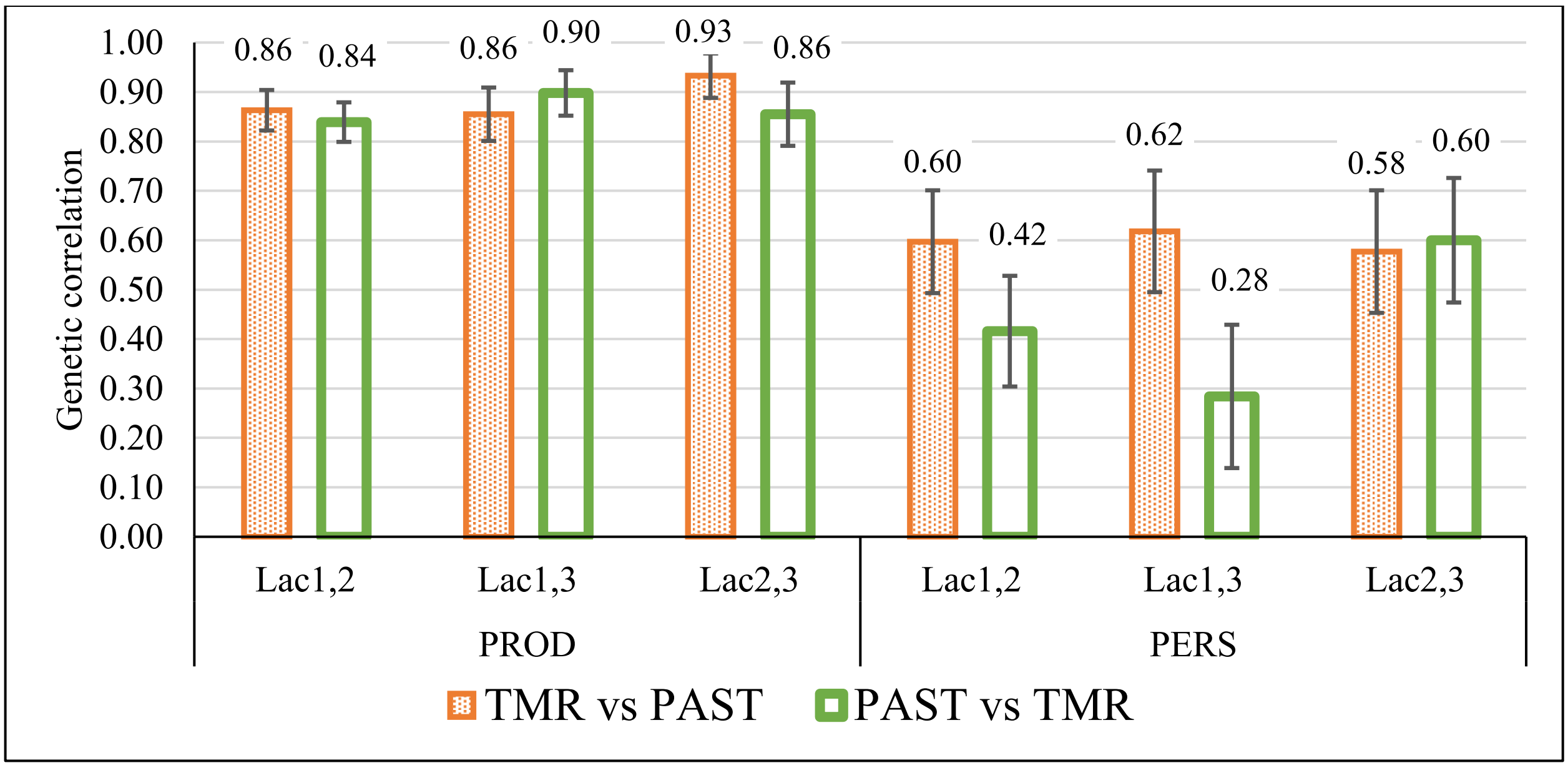

Laci,j = Lactation i,j; PROD = Production level; PERS = Persistency; TMR = Total Mixed Ration production system; PAST = Pasture production system

Figure S20 Genetic correlations (standard errors) between lactations for the total mixed ration vs the pasture production system for Protein production

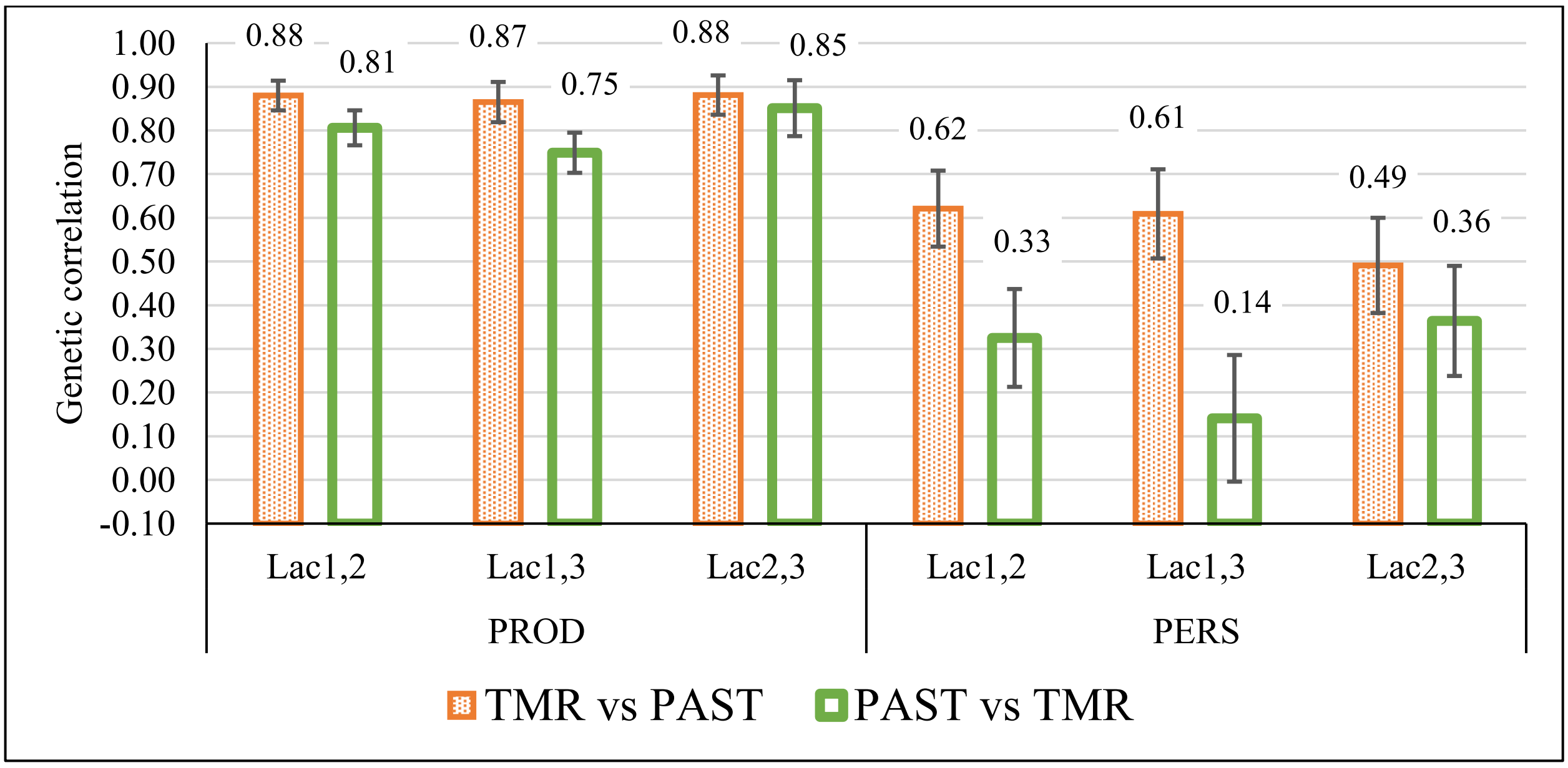

Laci,j = Lactation i,j; PROD = Production level; PERS = Persistency; TMR = Total Mixed Ration production system; PAST = Pasture production system
